# Animal models of interstitial cystitis/bladder pain syndrome

**DOI:** 10.3389/fphys.2023.1232017

**Published:** 2023-09-04

**Authors:** Cindy Tay, Luke Grundy

**Affiliations:** Neurourology Research Group, College of Medicine and Public Health, Flinders Health and Medical Research Institute, Flinders University, Adelaide, SA, Australia

**Keywords:** interstitial cystitis, bladder pain syndrome, IC/BPS, animal models, clinical translation

## Abstract

Interstitial Cystitis/Bladder Pain Syndrome (IC/BPS) is a chronic disorder characterized by pelvic and/or bladder pain, along with lower urinary tract symptoms that have a significant impact on an individual’s quality of life. The diverse range of symptoms and underlying causes in IC/BPS patients pose a significant challenge for effective disease management and the development of new and effective treatments. To facilitate the development of innovative therapies for IC/BPS, numerous preclinical animal models have been developed, each focusing on distinct pathophysiological components such as localized urothelial permeability or inflammation, psychological stress, autoimmunity, and central sensitization. However, since the precise etiopathophysiology of IC/BPS remains undefined, these animal models have primarily aimed to replicate the key clinical symptoms of bladder hypersensitivity and pain to enhance the translatability of potential therapeutics. Several animal models have now been characterized to mimic the major symptoms of IC/BPS, and significant progress has been made in refining these models to induce chronic symptomatology that more closely resembles the IC/BPS phenotype. Nevertheless, it's important to note that no single model can fully replicate all aspects of the human disease. When selecting an appropriate model for preclinical therapeutic evaluation, consideration must be given to the specific pathology believed to underlie the development of IC/BPS symptoms in a particular patient group, as well as the type and severity of the model, its duration, and the proposed intervention’s mechanism of action. Therefore, it is likely that different models will continue to be necessary for preclinical drug development, depending on the unique etiology of IC/BPS being investigated.

## 1 Introduction

Interstitial Cystitis/Bladder Pain Syndrome (IC/BPS) is a chronic disorder characterised by pelvic and/or bladder pain that is commonly reported with urinary urgency (Berry et al., 2011; Grundy et al., 2018a). However, significant heterogeneity exists in both the clinical symptoms and pathophysiological presentation of IC/BPS patients, presenting a major challenge to diagnosis, effective disease management, and the development of efficacious treatments. Consequently, IC/BPS is associated with a significant ongoing health burden, and a corresponding social and economic cost of greater than $20 Billion per annum in the United States (Pierce and Christianson, 2015).

To advance the understanding of IC/BPS and the development of novel therapeutics, numerous animal models have been developed that recapitulate the most common pathophysiological features of IC/BPS, including urothelial permeability, bladder inflammation, bladder/pelvic pain, and urinary frequency. However, clinical translation of preclinical research into novel and efficacious pharmacological treatments has been limited and there are still no effective long-term treatments for the debilitating symptoms of IC/BPS (Garzon et al., 2020a).

This review summarises current IC/BPS diagnosis and treatment options, the mechanisms thought to underlie IC/BPS pathophysiology, and the animal models available to investigate the pathophysiology and symptoms of IC/BPS. We discuss these models in the context of clinical relevance and offer insights into how these models can be used in future studies to increase our understanding of IC/BPS pathophysiology and advance the development of efficacious therapeutic strategies.

## 2 Epidemiology and clinical significance

IC/BPS affects approximately 4% of the population in western countries with a five times higher incidence in women than men (Jones and Nyberg, 1997; Berry et al., 2011; Pierce and Christianson, 2015). Patients with IC/BPS exhibit bladder-centric symptoms including urinary urgency and bladder pain at physiological bladder volumes (Kim et al., 2009; Grundy et al., 2019). The chronic nature of IC/BPS symptoms drastically diminishes quality of life, relentlessly impacting all aspects of personal and professional life, with ∼84% of IC/BPS patients finding employment or keeping a job difficult (Koziol et al., 1993; Tubaro, 2004; Dmochowski and Newman, 2007; Nickel et al., 2010; Berry et al., 2011; Bosch and Bosch, 2014; Vasudevan and Moldwin, 2017; Nunez-Badinez et al., 2021). As a result, psychosocial comorbidities are common in IC/BPS patients, with higher reported incidences of anxiety and depression that lead to a chronic decline in patient’s mental and physical health (Tubaro, 2004; Clemens et al., 2008; Chung et al., 2014; Nunez-Badinez et al., 2021; Ueda et al., 2021). Despite this burden, and decades of research, effective long-term treatments for IC/BPS are lacking (Garzon et al., 2020a). As a result, IC/BPS patients in the United States alone carry an economic burden of ∼$20–40 billion per annum (Pierce and Christianson, 2015). Therefore, there is an urgent need to develop effective treatments that improve the quality of life for IC/BPS patients.

## 3 Classification of IC/BPS

The American Urological Association defines IC/BPS as ‘An unpleasant sensation (pain, pressure, discomfort) perceived to be related to the urinary bladder, associated with lower urinary tract symptoms of more than 6 weeks duration, in the absence of infection or other identifiable causes’ (Hanno et al., 2011a).

Although IC/BPS patients present with common symptoms, including bladder pain and lower urinary tract symptoms, it is a heterogeneous clinical syndrome. Distinct subgroups or phenotypes exist that are categorised by highly divergent pathophysiology or responses to treatment. 5%–57% of IC/BPS patients have Hunner lesions (Whitmore et al., 2019; Akiyama et al., 2020), reddish mucosal lesions accompanied by abnormal capillary structures that are associated with more severe bladder inflammation and urothelial denudation (Peeker and Fall 2002; Logadottir et al., 2014; Jhang and Kuo, 2016a; Kim et al., 2017; Akiyama et al., 2018). Non-Hunner lesion IC/BPS patients exhibit less bladder inflammation (Peters et al., 2011; Warren, 2014; Whitmore et al., 2019), but commonly report more widespread symptoms and painful comorbidities including irritable bowel syndrome, fibromyalgia, and migraines indicative of a systemic syndrome (Jhang and Kuo, 2016a). Whilst the etiopathophysiology of IC/BPS is still unknown, it is increasingly likely that IC/BPS with Hunner lesions and IC/BPS without Hunner lesions have distinct pathophysiological origins (Fall et al., 2014; Maeda et al., 2015; Whitmore et al., 2019).

## 4 Diagnosis

Diagnosis for IC/BPS relies predominantly on the presence of chronic pelvic pain, which can include suprapubic pain, pressure or discomfort related to bladder filling and pain throughout the pelvis, in the absence of other definable disease (Hanno et al., 2011a; Hanno et al., 2015). As such, clinical diagnosis requires a comprehensive analysis of patient personal and medical history to rule out alternative sources of bladder pain and dysfunction such as medication, chemo- or radiotherapy induced cystitis, or neurological disorders associated with bladder dysfunction including spinal cord injury, stroke, Parkinson’s disease, and multiple sclerosis. Patients will also commonly undergo an abdominal and pelvic examination to exclude vaginitis and urethritis in addition to urinalysis and urine culture to exclude urinary tract infections, sexually transmitted infections, as well as malignancy of the bladder, uterus, vagina and ovaries (Hanno et al., 2015). Performing cystoscopy and urodynamic testing is not required for diagnosis, but can be performed to confirm the presence of Hunner lesions if the patient fits the relevant risk factors (Hanno et al., 2015). Cystoscopy is a necessary procedure in diagnosing IC/BPS based on East Asian guidelines (Ueda et al., 2021).

## 5 Mechanisms underlying IC/BPS

**FIGURE 1:**
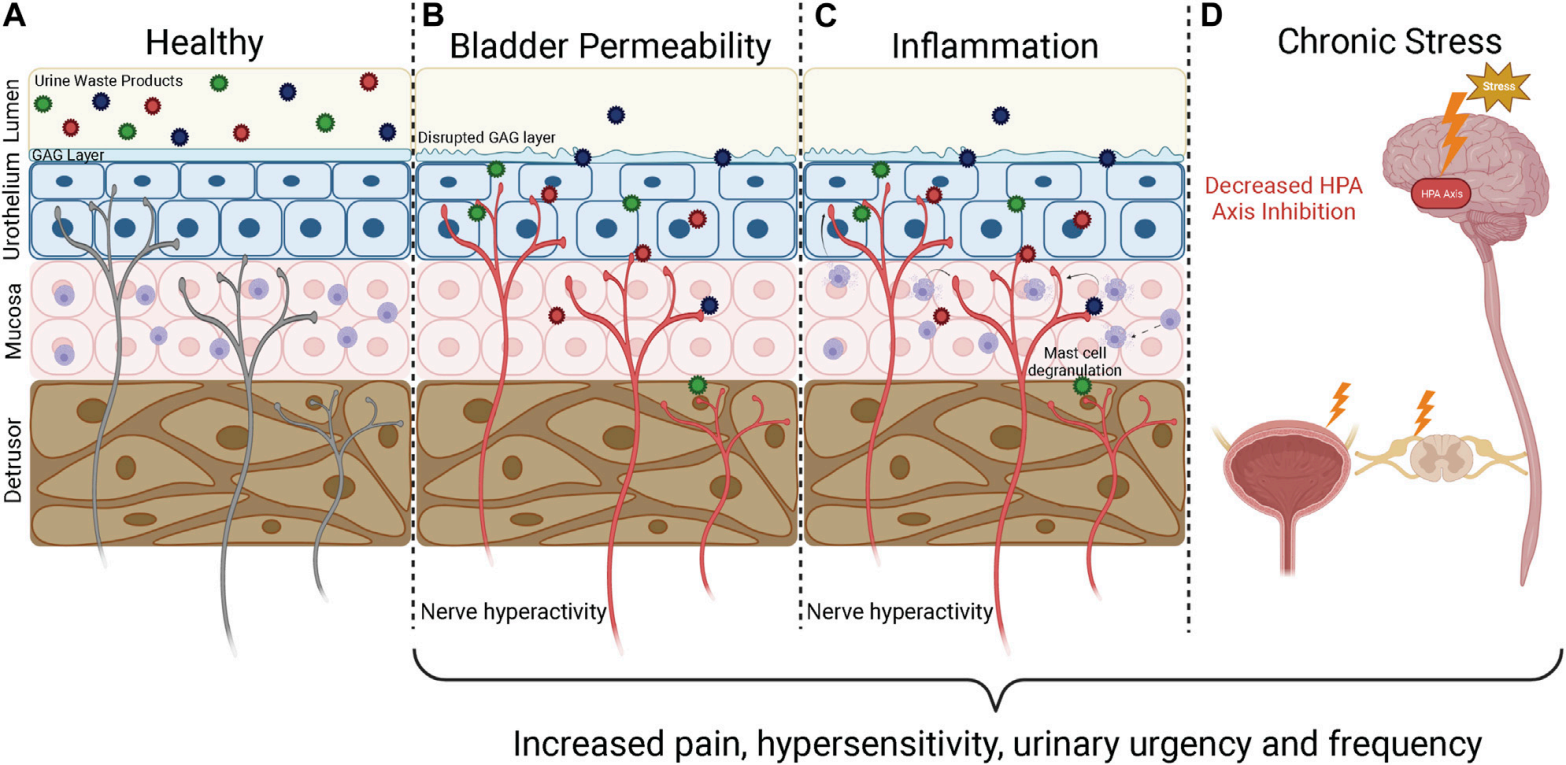
Underlying mechanisms of IC/BPS pathophysiology. The bladder wall is innervated by a dense network of afferent nerves that detect bladder stretch during bladder filling and danger signals generated during inflammation and barrier breakdown. If these sensory nerves become sensitised, they respond to physiological stimuli with greater intensity, leading to the generation of bladder hypersensitivity symptoms characteristic of IC/BPS including urinary urgency and pain. **(A)** In a healthy bladder the urothelium is impermeable, providing a barrier that prevents the toxic waste products contained within urine from accessing the bladder wall to activate the underlying sensory nerves. **(B)** An increase in bladder permeability due to breakdown of the urothelial barrier allows urine waste products to access the bladder interstitium. The influx of waste products causes further damage to the urothelium, increasing bladder permeability and allowing urine to reach deeper layers of the bladder to activate and sensitise bladder sensory nerve endings. **(C)** Persistent bladder permeability, bacterial infections, and autoimmunity trigger an inflammatory response within the bladder wall characterised by immune cell infiltration, mast cell degranulation, and the release of pro-inflammatory mediators including histamine and cytokines that can sensitise and activate nearby afferent nerve endings. Inflammation also enhances bladder permeability, allowing toxic waste products to enter the bladder and perpetuate the inflammatory state. **(D)** The HPA axis is responsible for regulating the stress response and provides input to the limbic system and prefrontal cortex to modulate sensory perception. Chronic HPA axis activation caused by severe stress leads to dysregulation of the HPA axis. HPA axis dysregulation can impact pain perception directly by modulating central nervous systems inputs and can lead to downstream effects on the spinal cord and hypersensitivity of bladder afferent nerves. GAG, Glycosaminoglycan; HPA, Hypothalamic Pituitary Adrenal. Figure was created using Biorender.com.

Bladder sensations arise following the activation of peripheral sensory afferent nerves embedded within the bladder wall, and the transmission of sensory signals into the central nervous system and brain where they can be processed and perceived (Fowler et al., 2008). Hypersensitivity of bladder-innervating afferents, such that exaggerated sensory signals are generated from the bladder during normal function, is considered a crucial component in the pathogenesis of IC/BPS symptoms (de Groat and Yoshimura, 2009; Grundy et al., 2018a). A variety of factors have been proposed to contribute to bladder afferent hypersensitivity in IC/BPS, including increased urothelial permeability, inflammation, and dysregulation of spinal and/or cortical networks (Figure 1) (de Groat et al., 2015; Grundy et al., 2018a). Despite the pathophysiology underlying afferent sensitisation being currently undefined, it is generally agreed that disruption of mucosal homeostasis, characterised by an increase in urothelial permeability and inflammation, is a major contributing factor to neuronal hypersensitivity and the painful symptoms of IC/BPS (Parsons, 2007; de Groat et al., 2015; Pierce and Christianson, 2015; Grundy et al., 2018a; Grundy et al., 2019; Karamali et al., 2019) (Figure 1). As such, unravelling the specific pathophysiological mechanisms involved in the development of neuronal hypersensitivity is likely to be critical to the development of novel therapeutics that effectively treat IC/BPS symptoms.

### 5.1 Increased bladder permeability

Urine contains a variety of toxic waste metabolites that are prevented from accessing the underlying bladder interstitium and sensory nerve endings by the usually impermeable urothelium. The urothelial barrier is maintained by tight junctions between apical urothelial cells, hydrophobic uroplakin plaques, and a considerable glycosaminoglycan (GAG) mucus layer of glycoproteins and proteoglycans that acts as a protective barrier between urine and urothelial cells (Jafari and Rohn, 2022; Klingler, 2016; Wyndaele et al., 2019). Several clinical studies have revealed that IC/BPS patients have a diminished or damaged urothelium (Elbadawi and Light, 1996; Tomaszewski et al., 2001; Keay et al., 2014; Hurst et al., 2015), providing toxic irritants and urea greater access to the cell membranes of urothelial cells (Figure 1). IC/BPS patients also have reduced expression of tight junction proteins, including E-cadherin and zonula-occludens-1 compared with healthy controls (Liu et al., 2012a; Jhang and Kuo, 2016a). A decrease in tight junction proteins allows urinary solutes to diffuse through the urothelium into the lamina propria to activate afferent nerve endings and precipitate urological symptoms consistent with IC/BPS (Davis et al., 2014). The urothelium can also be damaged further when in contact with high concentrations of cationic urinary components (Parsons et al., 2000; Parsons et al., 2014), allowing increasing amounts of urine to leak through to the deeper layers, exacerbating afferent hypersensitivity.

Whilst an increase in urothelial permeability clearly exaggerates bladder pain symptoms in IC/BPS, it is not yet known if urothelial permeability is a crucial component in the pathogenesis of bladder hypersensitivity in IC/PBS or a consequence of inflammation that acts to entrench a chronic disease state.

### 5.2 Inflammation

Inflammatory mediator sensitisation of afferent nerves is a pivotal component of the healing process, providing awareness of an injury to alter behaviour and promote tissue regeneration. However, if inflammation becomes uncontrolled this can be detrimental to tissue repair (Eming et al., 2007; Leoni et al., 2015; Landén et al., 2016), and can trigger long term changes in sensory afferent networks to induce a persistent hypersensitive state.

Only a minority of IC/BPS patients exhibit significant bladder inflammation and the development of Hunner’s lesions (Leiby et al., 2007; Whitmore et al., 2019). However, some degree of inflammation is common in the bladders of IC/BPS patients without Hunner lesions, with higher levels of pro-inflammatory mediators, including cytokines, chemokines, histamine, and nerve growth factor compared to healthy control bladders. IC/BPS bladders have also been shown to overexpress pro-inflammatory genes, exhibit mild oedema and tissue granulation, and have elevated numbers of immune cells, including mast cell, macrophages, eosinophils as well as T and B cell markers compared to healthy control bladders (Grundy et al., 2018a; Jhang and Kuo, 2016b; Peters et al., 1999; Liu and Kuo, 2012; Furuta et al., 2018; Liu et al., 2012a; Jacobs et al., 2010; el-Mansoury et al., 1994; Kastrup et al., 1983; Abernethy et al., 2017a; Hauser et al., 2008a; Sant et al., 2007; Grover et al., 2011; Hauser et al., 2008b; Abernethy et al., 2017a). Preclinical studies have confirmed that pro-inflammatory mediators can directly sensitise afferent nerve endings within the bladder wall (de Groat and Yoshimura, 2009; Hughes et al., 2013; Davidson et al., 2014; Grundy et al., 2020a; Grundy et al., 2021), providing a crucial link between inflammation and exaggerated sensation. Furthermore, it is well known that an inflammatory environment disrupts mucosal homeostasis and is detrimental to epithelial regeneration and repair during wound healing (Raziyeva et al., 2021). As such, localised inflammation within the bladder mucosa has the potential to increase bladder permeability, combining to establish a positive feedback cycle that further promotes an inflammatory state and chronic sensitisation of peripheral afferent endings within the bladder wall (Figure 1) (Sant et al., 2007; Grover et al., 2011; Grundy et al., 2018a). Whilst numerous inflammatory factors are elevated in the bladders of IC/BPS patients, whether these factors are a consequence of IC/BPS pathophysiology or contribute to the pathogenesis of IC/BPS in an otherwise healthy bladder has yet to be determined.

### 5.3 Chronic stress

Bladder sensory signals converge in the periaqueductal gray (PAG) of the midbrain with inputs from the limbic system (amygdala, hypothalamus, thalamus, cingulate gyrus), insula, and prefrontal cortex (Fowler et al., 2008). The hypothalamic pituitary adrenal (HPA) axis mediates the major adaptive component of the stress response and is a significant modulator of both the limbic system and sensory perception. Furthermore, bladder muscle function is under autonomic regulation, with stress imparting direct effects on bladder function. Modulation of the emotional affective state and homeostasis of the HPA axis can thus have overwhelming effects on bladder sensation and function and has been proposed as a key underlying mechanism in the development, persistence, and exacerbation of IC/BPS symptoms. In healthy patients, stress modulation of bladder sensation and function is commonly observed as urinary urgency during acutely stressful situations. However, in addition to the acute impacts of stress on the bladder, strong correlations exist between chronic stress and anxiety in the symptomology of IC/BPS as well as other visceral pain disorders such as irritable bowel syndrome (Pierce and Christianson, 2015; Moloney et al., 2016). Furthermore, acute and chronic stress can exacerbate urgency and the severity of pain in established IC/BPS patients (Koziol et al., 1993; Lutgendorf et al., 2000; Rothrock et al., 2001; Pierce and Christianson, 2015). With this in mind, chronic stress has been identified as a key risk factor in developing IC/BPS in otherwise healthy patients (Birder, 2019), and a number of studies have reported higher incidences of early life stress in IC/BPS patients than healthy controls (Fuentes and Christianson, 2018a). The precise mechanisms regulating stress induced IC/BPS are unclear, however, evidence is accumulating that the functional impacts of stress on bladder function and the perception of painful stimuli are likely mediated by long-term perturbations of the HPA axis and the sympathetic-adrenal medulla pathway (Figure 1) (de Groat et al., 2015; Fuentes and Christianson, 2018b; Bendrick et al., 2022). The downstream effectors of these pathways, including CRF, cortisol, and noradrenaline are well known regulators of urinary function and thought to be crucial in regulating centrally mediated changes that induce IC/BPS symptoms (Ulrich-Lai and Herman, 2009; Pierce and Christianson, 2015). Furthermore, clinical studies have revealed that chronic psychological stress induces heightened inflammatory responses in peripheral tissues, including elevated levels of circulating proinflammatory cytokines, and mastocytosis in the bladder (Charrua et al., 2015). Crucially, stress alleviation has been shown to be effective in reducing the severity of IC/BPS symptoms in some patients (Bosch and Bosch, 2014; Webster and Brennan, 1998; Carrico et al.).

## 6 Current available treatment for IC/BPS

Treatments for IC/BPS are delivered in a personalised and progressive manner in order of their invasiveness, potential to induce harmful side effects, and evidence for clinical success. We have summarised the clinical targets for each type of treatment (Table 1).

**TABLE 1:** The first-fourth line treatments available for IC/BPS.

Treatment type	Name of treatment	Target
Non-pharmacological Garzon et al. (2020a); Koziol et al. (1993); Lai et al. (2019); Hanno et al. (2015); Ueda et al. (2021); Ogawa et al. (2015); Davis et al. (2014)	Diet Modification	• Control voiding frequency
	Bladder Training	• Reduce bladder pain
Oral Medications Ogawa et al. (2015); Garzon et al. (2020a); van Ophoven et al. (2019); Taneja, (2021); Grigoryan et al. (2022)	Pentosan Polysulphate (PPS)	• Reduce urothelial permeability
		• Relieve bladder pain
		• Reduce urinary urgency
		• Reduce frequency of micturition
Intravesical Instillations Garzon et al. (2020a); Birder et al. (1997); Yoshimura et al. (2021); Tomoe, (2015); Grundy et al. (2018a); Parsons et al. (2015); Welk and Teichman, (2008); Parsons, (2005); Henry et al. (2015); Parsons et al. (2012); Henry et al. (2001); Nickel et al. (2009); Digesu et al. (2020)	Dimethylsulfoxide (DMSO)	• Smooth muscle relaxation
		• Blocks nerve activity
		• Provides anti-inflammatory effects
		• Relieve bladder pain and urinary frequency
	Lidocaine	• Blocks sensory nerve fibres in the bladder
		• Relieves bladder pain, urgency and nocturia
	Heparin	• Reproduce the activity of native bladder mucosa
		• Reduces transepithelial migration of solutes such as potassium that could depolarise sensory nerves to stimulate bladder pain, urgency and nocturia
Procedures Hanno et al. (2011a); McCahy and Styles, (1995); Glemain et al. (2002); Yamada et al. (2003); Ueda et al. (2021); Garzon et al. (2020a); Peters et al. (2007); Clemens et al. (2022a); Padilla-Fernandez et al. (2022); Hernández-Hernández et al. (2020)	Hydrodistension	• Increases bladder capacity to relieve urinary symptoms
		• Relives urinary urgency and frequency
		• Relieves bladder pain
	Neuromodulation	• Modulates neural pathways responsible for controlling bladder voiding
		• Relieves urinary urgency and frequency

### 6.1 Non-pharmacological treatments

Non-pharmacological treatments including diet and behavioural adaptations are initially offered to all patients to reduce symptom severity. Urine with an acidic pH is thought to exacerbate bladder irritation and can thus be harmful for IC/BPS patients with a diminished urothelium by increasing inflammation (Ueda et al., 2014; Ueda et al., 2021). Dietary modifications that exclude or limit certain foods such as citrus, coffee and alcohol can decrease urine pH to reduce bladder irritation (Koziol et al., 1993; Lai et al., 2019; Garzon et al., 2020b).

Behavioural adaptations incorporate a variety of modifications, including control of fluid intake, bladder training, and stress management. Bladder training is used to control urgency by incrementally and progressively increasing voiding intervals over 1–3 months (Davis et al., 2014; Hanno et al., 2015; Ogawa et al., 2015; Garzon et al., 2020b; Ueda et al., 2021). Bladder training is commonly employed for other urological disorders including overactive bladder syndrome and may be more useful in IC/BPS patients with mild/moderate symptoms (Chaiken et al., 1993; Foster et al., 2010). Patients are also encouraged to implement stress management practices, including increased exercise, as well as non-physical breathing/relaxation techniques, and psychotherapy if deemed necessary.

### 6.2 Oral medications

Pentosan polysulphate (PPS) is the only FDA approved treatment for IC/BPS (Garzon et al., 2020b; Ueda et al., 2021). PPS is a heparin-like agent that is intended to mimic glycosaminoglycans (GAG) within the bladder to restore urothelial impermeability (Ogawa et al., 2015; Garzon et al., 2020b). Meta-analyses of clinical trials using PPS have shown efficacy compared to placebo in providing moderate relief of bladder pain, urinary urgency, and frequency of micturition without significant side effects in specific subsets of patients (van Ophoven et al., 2019; Taneja, 2021; Grigoryan et al., 2022). However, long term use of PPS presents a risk of macular damage, vision-related injuries, gastrointestinal symptoms, and alopecia.

No new pharmacotherapies specifically designed for treating IC/BPS have been successfully developed, however, clinical data is now accumulating that repurposing immunosuppressive agents, such as Cyclosporine A (CyA) (Sairanen et al., 2004; Forrest et al., 2012; Ehrén et al., 2013; Crescenze et al., 2017), and Certolizumab Pegol (Bosch, 2018) may be efficacious in treating IC/BPS symptoms in patients refractory to approved oral and intravesical treatments. In particular CyA has been shown to have greater efficacy in patients with Hunner lesions, and the AUA now recommends oral CyA as fifth-line therapy for patients with Hunner lesions refractory to current treatments (Clemens et al., 2022a). Larger, longer, and multicenter randomized controlled trials are still required to further investigate certolizumab pegol as a treatment for IC/BPS. Whilst it is common for early-stage drug development not to translate into the clinic, there has been a significant and obvious lack of new oral medications for the treatment of IC/BPS. As a consequence, a variety of pre-existing medications have been trialled and are commonly prescribed in the hope of managing symptoms, including tricyclic antidepressants (amitriptyline), histamine receptor inhibitors (cimetidine and hydroxyzine) for which there is some evidence of efficacy (Henry Lai and Moldwin, 2017; Garzon et al., 2020b; Colemeadow et al., 2020).

### 6.3 Intravesical instillations

For those patients who do not respond to non-pharmacological or oral medications, intravesical instillations may be recommended.

Dimethylsulfoxide (DMSO) via temporary urethral catheter is an FDA-approved treatment for IC/BPS (Garzon et al., 2020b; Ueda et al., 2021), however, the optimal dwell time, length of induction therapy or length of maintenance therapy is unknown. DMSO induces smooth muscle relaxation, blocks sensory nerve activity, and is anti-inflammatory, and has been used effectively to relieve pain and urinary frequency in IC/BPS patients (Birder et al., 1997; Garzon et al., 2020b). DMSO is especially beneficial for IC/BPS with Hunner lesions (Tomoe, 2015; Yoshimura et al., 2021), however, a large proportion of patients relapse within 2 months of treatment.

Lidocaine/heparin: Lidocaine is a local anaesthetic that blocks voltage gated sodium channels present on the peripheral ends of bladder-innervating sensory nerves (Grundy et al., 2018c). Alkalinisation of lidocaine with sodium bicarbonate increase absorption via the urothelium and increases absorption into the neuronal cytoplasm to enhance the therapeutic effect. The inclusion of heparin within the infusion formulation, a naturally occurring glycosaminoglycan, is considered to provide additional benefits to the treatment of IC/BPS by restoring urothelial impermeability (Parsons et al., 1994). Clinical trials of intravesical instillation of lidocaine/heparin show efficacy in relieving IC/BPS symptoms (Henry et al., 2001; Parsons, 2005; Welk and Teichman, 2008; Nickel et al., 2009; Parsons et al., 2012; Henry et al., 2015; Parsons et al., 2015; Digesu et al., 2020), however, an optimal formulation of combined lidocaine and heparin has not been agreed upon, and its widespread use is limited by the requirement for urethral catheterisation.

### 6.4 Procedures

If behavioural, oral pharmacology, and intravesical instillations are unsuccessful at controlling symptoms, patients may be recommended for more invasive procedures including bladder hydrodistension or neuromodulation.

Hydrodistension of the bladder under high pressure (60–80 cm H_2_O) for a short duration (less than 10 min) can offer relief from urinary symptoms in 30%–55% of patients. However, symptom improvement decreases over time, requiring repeated procedures after only a few months (Hanno et al., 2011a; Garzon et al., 2020b; Ueda et al., 2021).

Neuromodulation has not been FDA-approved as a treatment for IC/BPS but has recently been clinically approved for select patients who have success in a nerve stimulation trial (Clemens et al., 2022b). Neuromodulation can lead to control over urinary symptoms through the emission of electrical stimulation that targets nerve activity due to bladder filling (Intern ational Neuromodulation Society, 2013; Padilla-Fernandez et al., 2022). There are two main neuromodulation techniques that are currently being explored to treat IC/BPS; sacral nerve stimulation and pudendal nerve stimulation (Clemens et al., 2022b; Padilla-Fernandez et al., 2022).

Sacral nerve stimulation involves the implantation of a generator under the skin and in the upper buttock area. A small electrode is also placed near the sacral nerve, which will receive electrical impulses from the neurotransmitter, that controls voiding function in the lower spine (Hernández-Hernández et al., 2020; International Neuromodulation Society, 2021a). Pudendal nerve stimulation is seen as an alternative method to sacral nerve stimulation. Similarly, the generator is placed in the upper buttock area, but the electrode is implanted near the pudendal nerve (Peters et al., 2007). The impulses from the generator will stimulate the pudendal nerve and control the pelvic floor muscle during bladder filling (International Neuromodulation Society, 2021b).

Both sacral and pudendal nerve stimulation has been shown to improve urinary symptoms, including bladder capacity, urinary frequency, voided volume, nocturia and pain (Peters et al., 2007; Clemens et al., 2022b; Padilla-Fernandez et al., 2022). However, only a small number of patients have been studied and there is a lack of evidence to suggest that neuromodulation is effective for long periods of time. The AUA guidelines state that sacral/pudendal neuromodulation may be effective in carefully selected patients and emphasise that the procedure can improve frequency/urgency symptoms but is less effective for pelvic pain (Clemens et al., 2022b).

### 6.5 Issues with available treatments

Despite the availability of multiple treatment options for IC/BPS, no currently available treatment has been shown to permanently reverse disease symptoms, and many patients remain refractory to treatment. As a result, patients continue to suffer with symptoms indefinitely, with available treatments generally only providing temporary relief of chronic pain or are sufficient in a sub-population of patients. At the time of writing, there are 30 clinical trials recruiting or active for interstitial cystitis (ClinicalTrials.gov, 2023), however, the only pharmacological tool being tested is the opioid antagonist Naltrexone (NorthShore University, 2022; Stanford, 2023).

Developing novel and efficacious treatments for IC/BPS is extremely challenging. The diversity of symptoms means that patients may need to take multiple medications or engage in additional interventions that target distinct symptomology. Furthermore, the lack of a defined pathophysiology means that the origin of IC/BPS symptoms may be highly distinct between patients. These clinical challenges are also replicated preclinically, with the diversity of disease and symptoms translating into a difficulty in establishing animal models that can faithfully recapitulate the full spectrum of IC/BPS pathophysiology and symptoms.

## 7 Animal models of IC/BPS

A key step in identifying novel therapeutic targets for a disease is being able to accurately mirror the human condition in an animal model, which allows determination of the pathological mechanisms that drive symptoms and the subsequent testing of novel therapeutics for symptom alleviation. Unfortunately, because myriad pathophysiological mechanisms have been proposed to mediate the development of bladder dysfunction and bladder hypersensitivity in IC/BPS, this has made the establishment of accurate animal models and the development of efficacious therapies for these disorders extremely challenging.

**FIGURE 2:**
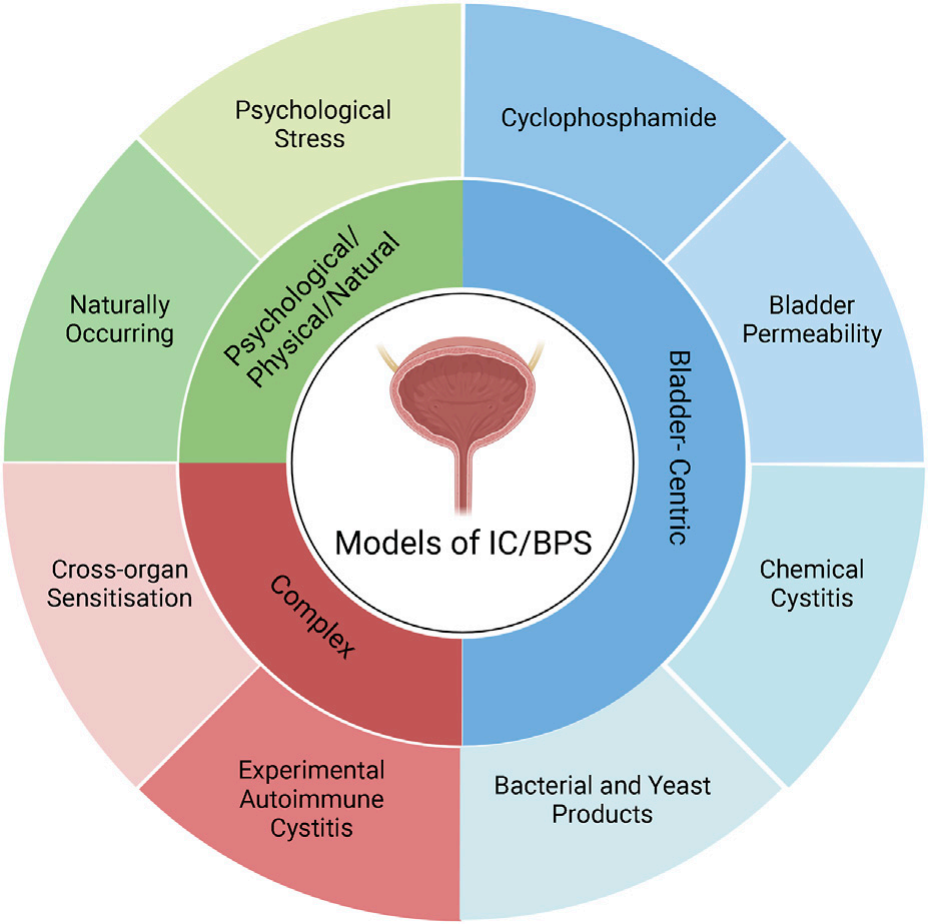
Classification of IC/BPS animal models. IC/BPS animal models can be broadly categorised into three different types: Bladder-centric models, models with complex mechanisms, and psychological and physical stressors/natural disease models. Bladder centric models induce an IC/BPS phenotype by direct insult to the bladder that recapitulates the inflammatory or bladder permeability pathophysiology of IC/BPS. Bladder centric models can be further stratified by the type of insult and/or the stimuli used and are the most utilised animal models of IC/BPS. Psychological/Physical/Natural models either have a naturally occurring IC/BPS phenotype such as feline interstitial cystitis or cause IC/BPS like symptoms via psychological stress that models the contribution of stress to the development of IC/BPS. Complex models of IC/BPS employ indirect interventions to generate an IC/BPS phenotype including cross-organ sensitisation from the colon and experimental autoimmune cystitis which have both been implicated in the pathophysiology of IC/BPS. Figure was created using Biorender.com.

**FIGURE 3:**
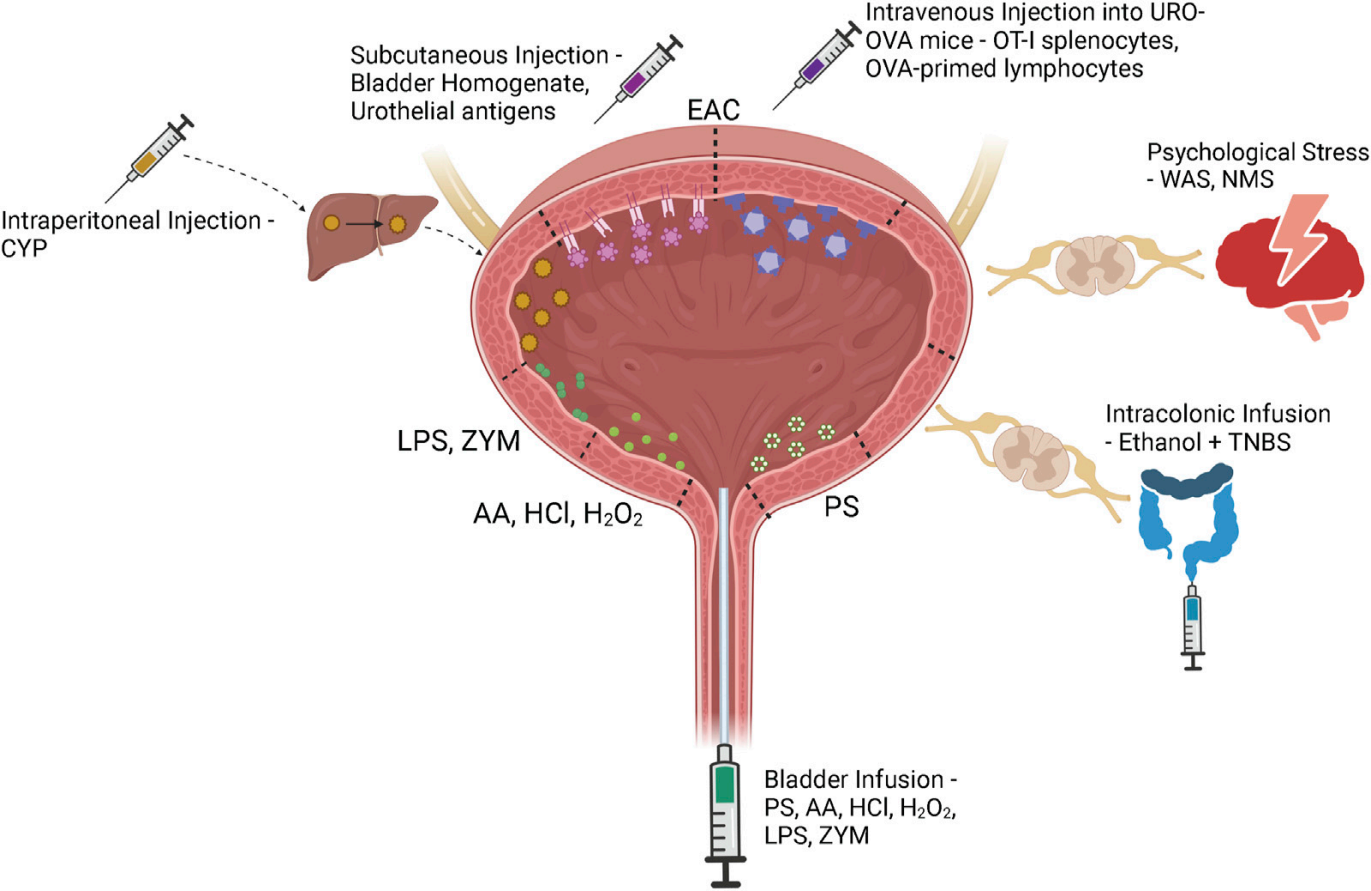
IC/BPS model induction methods. Bladder centric models including bladder permeability, chemical cystitis and bacterial and yeast models are induced by direct intravesical instillation of protamine sulfate (PS), acetic acid (AA), hydrochloric acid (HCl), hydrogen peroxide (H_2_O_2_), lipopolysaccharide (LPS), or zymosan (ZYM) via a bladder catheter. Although cyclophosphamide (CYP) is injected intraperitoneally into the animal, it is metabolised to acrolein in the liver and excreted in the urine to induce bladder damage and inflammation. Experimental autoimmune cystitis (EAC) models can be generated by subcutaneous injection of bladder homogenate or urothelial antigens that trigger autoimmunity through interactions with the membrane expressed MHC Class II molecules. The transgenic EAC model utilises urothelium-ovalbumin (URO-OVA) mice that express an OVA ‘self’ antigen on the membrane of urothelial cells. When OT-I splenocytes or OVA-primed lymphocytes are intravenously injected into URO-OVA mice they interact with the OVA antigen and trigger an autoimmune response. Psychological stress models including water avoidance stress (WAS) and neonatal maternal separation (NMS) induce bladder hypersensitivity via chronic stress induced dysregulation of the hypothalamic-pituitary-adrenal axis. Continued exposure to a stressful environment can deteriorate the animal’s stress response, affecting downstream bladder activity. Cross-organ sensitisation models are induced via intracolonic infusion of ethanol and TNBS. Colonic inflammation sensitises colonic afferents which induces bladder afferent hypersensitivity through viscero-visceral crosstalk between overlapping sensory networks. Figure was created using Biorender.com.

A variety of animal models have been developed to recapitulate the complex pathophysiology of IC/BPS (Figure 2). However, as the pathophysiology of IC/BPS is yet to be fully defined, and consists of numerous subclassifications, animal models have focussed primarily on establishing the defining symptoms of bladder hypersensitivity and pain utilising a variety of different methods (Figure 3). The following sections summarise the diverse range of currently utilised animal models and review the ability of these models to resemble distinct aspects of IC/BPS as well as their strengths and limitations (Table 2). The advantages, disadvantages and clinical relevance of each model has been summarised in Table 3.

**TABLE 2:** Summary of key characteristics of IC/BPS animal models.

Model	Species	Technique	Acute/Chronic dosing	Bladder damage	Inflammation	Permeability	Nociception	Voiding frequency
Protamine Sulphate Instillation Lavelle et al. (2002); Meerveld et al. (2015); Soler et al. (2008); Offiah et al. (2017); Grundy et al. (2020a); Stemler et al. (2013); Soler et al. (2008); Lasič et al. (2015); Hurst et al. (2015); Shin et al. (2011)	Female Mice and Rats	Bladder Instillation	Acute (single dose)	• Low doses—mild urothelial damage	• High doses: Infiltration of neutrophils into mucosa at higher doses	• Low dosage: Increase in transcellular permeability that returns to normal over 3–7 days	• Increased bladder afferent peak firing and decreased activation thresholds to bladder distension that returned to baseline by 7 days post infusion	• Increased number of contractions and total contraction time and decreased micturition threshold that returned to baseline after 7 days
				• Higher doses—develop urothelial ulceration and infiltration of neutrophils into the mucosa (dependent on the presence of urine)			• Significantly blunted VMR to bladder distension at noxious bladder distension pressures that returned to control levels by 7 days post treatment	
Cyclophosphamide Auge et al. (2013); Juszczak et al. (2010); Boucher et al. (2000); Smaldone et al. (2009); Chen et al. (2020); Okinami et al. (2014); Malley and Vizzard, (2002); Hu et al. (2003); Chopra et al. (2005); Coelho et al. (2015); Yoshimura and de Groat, (1999); Mills et al. (2020); Yang et al., (2021); Dang et al. (2008); Boudes et al. (2011), Boudes et al. (2013); DeBerry et al. (2014); Sugino et al. (2015); DeBerry et al. (2015); Gao et al. (2015); Auge et al. (2020); Yoshimura et al. (2021)	Male and Female Mice and Rats	IP injection	Acute (single 150–200 mg/kg dose) results taken within 1 day	• Thick bladder wall	• Infiltration of inflammatory cells	• Increased urothelial permeability to water and urea	• Decreased nociceptive threshold in response to innocuous stimulation with von-Frey hairs	• Increased non voiding contractions
				• Mucosal erosion on the luminal surface of the urothelium				• Decreased ICI
				• Severe oedema				
				• Redness				
				• Ulceration				
				• Haemorrhage				
			Chronic (40–100mg//kg every 2–3 days for 7 or 10 days)	• Extensive mucosal erosion	• Increased number of inflammatory cells	• Bladder permeability experiments have not been performed in this model	• Exaggerated EMG responses to noxious bladder distension	• Increased voiding frequency
				• Ulceration				
				• Oedema				
				• Petechial haemorrhages	• Upregulation of inflammatory cytokines		• Increased pERK immunoreactivity in the LS dorsal horn following bladder distension	• Decreased voided volume
				• Irregular ectasia vessels				
								• Reduced ICI
Chemical Cystitis Hauser et al. (2009); Song et al. (2017); Dogishi et al. (2017); Sahiner et al. (2018); Danacioglu et al. (2021); Kirimoto et al. (2007); Song et al. (2015); Çayan et al. (2003); Fraser et al. (2003)	Female Mice	Bladder Instillation	Acetic acid	• Urothelial thinning and cellular loss or erosion after 14 days	• No reported changes in inflammatory response	• Bladder permeability experiments have not been performed in this model	• Nociception experiments have not been performed on this model	• Reduced ICI compared to controls that persisted for up to 7 days
			Acute					
			Hydrochloric Acid (HCl) Acute	• Urothelial thinning and cellular loss or erosion after 14 days	• Infiltration of chronic inflammatory cells (eosinophils, mast cells)	• Bladder permeability experiments have not been performed in this model	• Nociception experiments have not been performed on this model	• Irregular voiding frequency
				• Lesions in the epithelium and lamina propria				• Decreased inter-contraction interval for up to 7 days
				• Oedema				• Decreased voided volume
				• Thickening of the transitional epithelium				• Smaller bladder capacity
				• Fibroblast swelling				
			Hydrogen Peroxide (H_2_O_2_) Acute	• Haemorrhage, oedema and urothelium denudation observed after 1, 7 and 14 days	• Large number of neutrophils and mast cells from 1 to 14 days	• Bladder permeability experiments have not been performed in this model	• Nociception experiments have not been performed on this model	• Significantly more frequent micturition events
				• Vascularisation of the lamina propria observed after 7 and 14 days	• Severe neutrophilic and mononuclear infiltration after 7 days			• Decreased voided volume after 1 day
				• Eventual hyperplasia by 7 and 14 days due to thickening of urothelium	• Small number of infiltrated eosinophils and lymphocytes on 7 and 14 days			• Significantly lower ICI
Zymosan Randich et al., (2006b), Randich et al., (2006a); DeBerry et al., (2007); Clodfelder-Miller et al., (2022); Ramsay et al., DeBerry et al., (2010); Randich et al., (2009); Ness et al., (2021); Liu et al., (2021)	Female—Rats (neonates for chronic), mice and guinea pigs	Bladder Instillation	Acute	• Increased mucosal thickness	• Increased white blood cells within bladder wall observed 1 day after induction	• Enhanced bladder permeability 1 day post infusion	• Enhanced VMR to UBD 1 day post infusion	• Increased voiding frequency
			(Single dose of zymosan + protamine sulfate (guinea pigs))				• Bladder hypersensitivity of mucosal afferents to mucosal stroking and high threshold muscular afferents to bladder stretch	• Decreased voided volume
			Chronic	• No histological abnormalities/damage between zymosan and controls	• Inflammatory response has not been comprehensively characterised.	• Increased neurogenic plasma extravasation in the bladder as adults	• Bladder hypersensitivity to infusion of ice-cold saline	• Significantly more micturition events
			(Repetitive zymosan instillation in neonatal rats)		• Overall reports suggest it is amongst the mildest of bladder-centric models		• Enhanced VMR to bladder distension	• Decreased micturition volumes
								• Decreased micturition volume thresholds
								• Increased micturition frequency as adults
Bacterial Products—Lipopolysaccharide (LPS)	Female—rats and mice	Bladder Instillation + protamine sulfate	Acute	• Oedema and haemorrhage	• Infiltration of mononuclear and polymorphonuclear leukocytes and neutrophils 1 day post LPS	• Bladder permeability experiments have not been performed in this model	• Nociception experiments have not been performed in this model	• Altered bladder voiding behaviour
			(Single LPS instillation)	• Vacuolisation of urothelial cells	• Mast cell infiltration up to 5 days after infusion			• Increased intra-bladder pressure in response to bladder filling and shorter ICI at 1–3 days after LPS instillation
								• Higher micturition frequency
					• Increased expression of pro-inflammatory cytokines			• Lower maximum pressure
Jerde et al. (2000), Lv et al. (2012), Ryu et al. (2019), Yoshizumi et al. (2021), Li et al. (2017), Sinanoglu et al. (2014), Saban et al. (2002), Tambaro et al. (2014), Song et al. (2017), Raetz and Whitfield (2002), Bjorling et al. (2011)			Chronic	• Severely compromised urothelium leading to bladder remodelling	• Severe inflammatory cell infiltration of macrophages, lymphocytes and mast cells	• Bladder permeability experiments have not been performed in this model	• Increased bladder hypersensitivity after 7, 14 and 21 days	• Shorter voiding intervals (7 days)
			(Multiple LPS instillations across several weeks)	• Increased urothelial cells	• Enhanced urinary cytokine concentrations		• Significantly decreased withdrawal thresholds in the abdomen and hind paw	• Increased non voiding contractions
				• Abnormally thick re-epithelialisation and tissue fibrosis				• Decreased bladder capacity and significantly decreased peak and threshold pressures
Feline Interstitial Cystitis Mohamaden et al. (2019); Jones et al. (2021); Kruger et al. (1991), Kruger et al. (2009); Defauw et al. (2010); Lulich et al. (2010); Lavelle et al. (2000); Roppolo et al. (2005); Birder and Andersson, (2013)	Felines	Naturally occurring	Chronic	• Thinning and denudation of the urothelium	• Infiltration of lymphocytes and inflammatory cells in the bladder interstitium	• Significantly reduced transepithelial resistance of the urothelium	• Bladder Aδ afferents from FIC cats are hypersensitive to bladder distension	• Voiding experiments have not been performed in this model
				• Urothelial spongiosis—loss of cell-cell adhesion	• Increased mast cells	• Water and urea permeability significantly increased		
				• Tight junctions come apart	• Significantly higher serum concentrations urinary cytokines	• Lower expression of E-cadherin tight junction protein		
Experimental Autoimmune Cystitis Jin et al. (2017); Lin et al. (2008); Liu et al. (2019); Singh et al. (2013); Izgi et al. (2013); Altuntas et al. (2012); Bicer et al. (2015); Liu et al. (2007); Akiyama et al. (2021); Cui et al. (2019); Kim et al. (2011); Kullmann et al. (2018); Wang et al. (2016)	Female Mice	Urinary bladder Homogenate	Chronic	• Submucosal oedema	• Increased levels of cytokines, chemokines, mast cells, neutrophils and infiltration of CD4^+^ lymphocytes 14 days from second immunisation	• Bladder permeability experiments have not been performed for this model	• Hyperalgesia to von Frey hair probing of the pelvic area and decreased pelvic pain threshold 14 days after second immunisation	• Shorter voiding intervals
		(Subcutaneous injection of bladder homogenates)		• Urothelial detachment from the lamina propria				• Decreased voided volume
				• Thickening of the lamina propria 28 days from first immunisation				• Increased number of urine spots
		Uroplakin	Chronic	• Bladder remodelling	• Increased gene expression of inflammatory cytokines	• Bladder permeability experiments have not been performed for this model	• Greater sensitivity to von Frey probing of the suprapubic region from 5 to 40 days after immunisation	• Altered bladder function developed 35 days after immunisation
					• Extensive perivascular leukocyte			• Increased urinary frequency
		(Subcutaneous injection of recombinant mouse uroplakin proteins)			• Higher expression of the mast cell chemoattractant/activator CCL2			• Decreased mean urine outputs per void
					• Increased numbers of activated, resting and total mast cells in the bladder detrusor at 10, 20 and 40 days after immunisation			
		Transgenic URO-OVA Models	Chronic	• Interstitial oedema	• Mononuclear cellular infiltration—T (CD3^+^) and B (CD19^+^) lymphocytes	• Bladder permeability experiments have not been performed for this model	• Significantly increased pelvic nociceptive responses to von Frey hairs	• Altered voiding behaviours developed 7–28 days after cystitis induction
		(Intravenous injection of activated OVA-specific T-cells into URO-OVA mice)		• Increased vascularity	• Mucosal hyperemia		• Significantly decreased sensory thresholds to pelvic nociception	• Decreased maximum volume voided per micturition
				• Epithelial hyperplasia lasts for 7–28 days after adoptive transfer	• Increased mRNA expression of mast cell and sensory neuron-derived inflammatory factors		• Increased VMR to bladder distension	• Significant increase in the frequency of urination
					• 2-fold increase in mast cells within the lamina propria and the detrusor			
Psychological Stress Models Pierce et al. (2018), Pierce et al. (2016); West et al. (2021); Matos et al. (2017); Lee et al. (2015); Gao et al. (2018); Wang et al. (2017); Robbins et al. (2007); Dias et al. (2019)	Male and Female Rats and Mice	Water Avoidance Stress Models	Chronic	• Loss of superficial umbrella cells	• Increased inflammatory cells infiltration in the mucosa	• Bladder permeability experiments have not been performed for this model	• Significantly increased frequency of responses to von-Frey hairs at 5 days	• Significant increase in urinary frequency
		(Rats placed on a platform in the middle of a tank filled with water—performed 1h a day over 10 days)		• Altered urothelial surface	• Higher number of mast cells		• Reached a plateau after 8 days	• Decrease in the average void size
							• VMR evoked at lower bladder pressure and for a longer duration	• Increase in the number of small voids by 3 days of WAS exposure
		Neonatal Maternal Separation (Separation of litters of pups from the dam for up to 21 days from postnatal D1)	Chronic	• Bladder damage has not been studied for this model	• Lower mRNA levels of CRF_1_ and GR and higher BDNF in the hippocampus	• Bladder permeability experiments have not been performed for this model	• Greater VMR response to UBD after 56 days	
					• Low CRF and GR levels indicate decreased inhibition on the HPA axis			• Increased voiding frequency
					• Decreases animal resilience to stress over time			• Smaller void spots
					• Significantly higher percentage of degranulate mast cells in the bladder			
Cross-organ Sensitisation Models Antoniou et al. (2016); Hughes et al. (2009); Vannucchi and Evangelista, (2018); Grundy and Brierley, (2018); Grundy et al. (2018a); Meerveld et al. (2015); Towner et al. (2015); Lei and Malykhina, (2012); Xia et al. (2012); Fitzgerald et al. (2013); Ustinova et al. (2007); Liang et al. (2007); Lamb et al. (2006); Ustinova et al. (2006)	Male and Female Rats and Mice	Intracolonic co-administration of ethanol and TNBS	Chronic	• No changes in bladder histology	• No marked bladder inflammation	• Urothelial permeability increases during the active phase of colonic inflammation from 1 to 7 days post TNBS	• Bladder sensory nerves exhibit hypersensitivity to UBD during the active inflammatory phase of TNBS colitis and 28 days post colitis	• Reduced bladder capacity, voided volumes, ICI and changes in bladder voiding patterns persist up to 90 days post TNBS
							• Enhanced bladder VMR to UBD	

**TABLE 3:** Advantages, disadvantages and clinical relevance of each IC/BPS model.

Model	Advantage	Disadvantage	Clinical relevance
Bladder Permeability Models with Protamine Sulphate	• Results in bladder permeability• Bladder damage dependent on the presence of urine supports concept well established leaky urothelium to bladder inflammation pathomechanism	• Can only be performed in female rodents due to catheterisation	• Recapitulates only limited aspects of the IC/BPS phenotype
		• Shows limited inflammation	
		• No reported changes in bladder function	
		• Inconsistent reports of alterations of bladder sensation or sensory signalling	
		• Also supports pathophysiology that inflammation is necessary to maintain urothelial permeability generating a feedback loop	
Cyclophosphamide (CYP)	• Can be easily done in both male and female animals	• Results in a relatively transient effect on the bladder and does not recapitulate the chronic and progressive nature of IC/BPS	• This best models IC/BPS without Hunner lesions.
	• Model is well established and supported by a large amount of literature		
	• Animals develop increased voiding frequency and bladder hypersensitivity which are key hallmarks of IC/BPS	• Results in a relatively transient effect on the bladder and does not recapitulate the chronic and progressive nature of IC/BPS	• Clinically relevant to patients who develop post-chemotherapy induced cystitis
	• Animals also develop bladder damage and infiltration which mostly mirror clinical observations from IC/BPS patients		
Chemical Cystitis—using Acetic Acid (AA), Hydrogen chloride (HCl) and Hydrogen Peroxide (H_2_O_2_)	• Effects are bladder-centric and there are no confounding impacts on the body	• Can only be performed in female rodents	• Extremely severe bladder damage that seems to be more characteristic of IC/BPS with Hunner lesions
	• Has a long lasting effect and develop into chronic changes in bladder function	• Results in severe bladder inflammation that exceeds IC/BPS without Hunner lesions • No reports on whether animals develop bladder hypersensitivity	
	• Model results in altered voiding behaviours		
Zymosan	• Dual insult version of this model recapitulates IC/BPS patients with previous bladder infection well	• Can only be performed in female rodents due to catheterisation	• Chronic model of IC/BPS without Hunner lesions
	• After second inflammatory insult, adult mice develop greater bladder hypersensitivity and altered voiding behaviour	• No reports of developed bladder damage in chronic models	• Clinically relevant to IC/BPS patients who have previously had an early life bladder infection
Bacterial Products (Lipopolysaccharide)	• Developed chronic model that results in urothelial denudation, tissue fibrosis and infiltration of inflammatory cells and cytokines	• Can only be performed in female rodents due to catheterisation	• The chronic model results in a severely compromised urothelium that eventually results in bladder remodelling and tissue fibrosis as well as mast cell infiltration which are characteristics of IC/BPS without Hunner lesions
	• Animals also developed altered voiding behaviours and bladder hypersensitivity	• Results in severe bladder damage	
Naturally Occurring—Feline Interstitial Cystitis	• Model does not require external intervention	• Natural occurring in felines where the aetiology is unknown	• While there are many shared phenotypes between FIC and IC/BPS without Hunner lesions, the underlying cause for either disease is still unknown, therefore unclear whether FIC is a reliable model of IC/BPS
	• Development of histological features—urothelial denudation, submucosal oedema, chronic inflammatory cell infiltrates and muscularis fibrosis	• Model is not widely available—difficult to find reasonable numbers of cats for experimental purposes	
	• Bladder afferents from FIC cats become hypersensitive to bladder distension	• Ethical considerations with studying cats for more in depth bladder activity—organising and consulting with a veterinarian and a higher cost of maintenance	
Urinary bladder homogenate	• Can be performed in C57BL/6J mice which are widely used and also express IA^B^ MHB class II molecules that are identical to human beings	• Induces non-specific immune response as bladder homogenate is not a tissue-specific protein for immunisation	• Model closely represents IC/BPS with Hunner lesions which has been hypothesised to have an autoimmune nature
	• Animals developed bladder damage, inflammation, increased urinary frequency and bladder hyperalgesia		
Uroplakin	• Models exhibit extensive bladder damage and inflammation	• Not performed in C57BL/6J mice—less accessible	• Model closely represents IC/BPS with Hunner lesions which has been hypothesised to have an autoimmune nature
	• Developed increased urinary frequency	• UPK3A 65–84 is a specific peptide to induce autoimmunity in BALB/c mice	
	• UPK3A 65–84 mice showed bladder hypersensitivity		
Transgenic URO-OVA	• Animals developed bladder inflammation	• OVA is not an endogenous antigen of bladder	• Model closely represents IC/BPS with Hunner lesions which has been hypothesised to have an autoimmune nature
	• Some bladder damage but not urothelial denudation	• Have been used only in female mice so far	
	• Had increased urinary frequency		
	• Exhibited bladder hypersensitivity		
Water Avoidance Stress	• Animals develop altered voiding behaviour and bladder hypersensitivity	• Gut and bladder interact in health and disease and therefore delineating the direct from indirect effects of WAS on bladder function are difficult	• Model represents patients with chronic stress and anxiety who develop IC/BPS without Hunners lesions
	• Loss of superficial umbrella cells leading to an altered urothelial surface	• Majority of effects on bladder function have only been characterised at relatively short intervals	
	• Signs of increased inflammatory cells infiltration in the mucosa		
Neonatal Maternal Separation (NMS) Models	• Can be performed in both male and female animals	• No disadvantages found	• Models the high proportion of IC/BPS without Hunner lesions patients that also have comorbid anxiety and or history of psychological trauma
	• Does not cause significant inflammation in the bladder		
	• Induces long lasting sensory hypersensitivity		
	• Induces long lasting bladder hyperactivity		
	• Induces mild bladder permeability		
	• NMS has been shown to have effects on brain structures affecting HPA axis signalling		
Cross-organ sensitisation	• Does not cause significant inflammation in the bladder	• No reports of bladder inflammation	• Models the high proportion of IC/BPS patients that also have chronic abdominal pain, IBS
	• Induces long lasting sensory hypersensitivity		
	• Induces long lasting bladder hyperactivity		
	• Induces short-lasting bladder permeability		
	• Can be performed in both male and female animals		
	• Relatively simple to establish (inexpensive)		

### 7.1 Urothelial permeability models

Despite the wealth of clinical evidence supporting a role for increased urothelial permeability in the pathophysiology of IC/BPS, there are relatively few animal models that exclusively target this pathophysiology.

**FIGURE 4:**
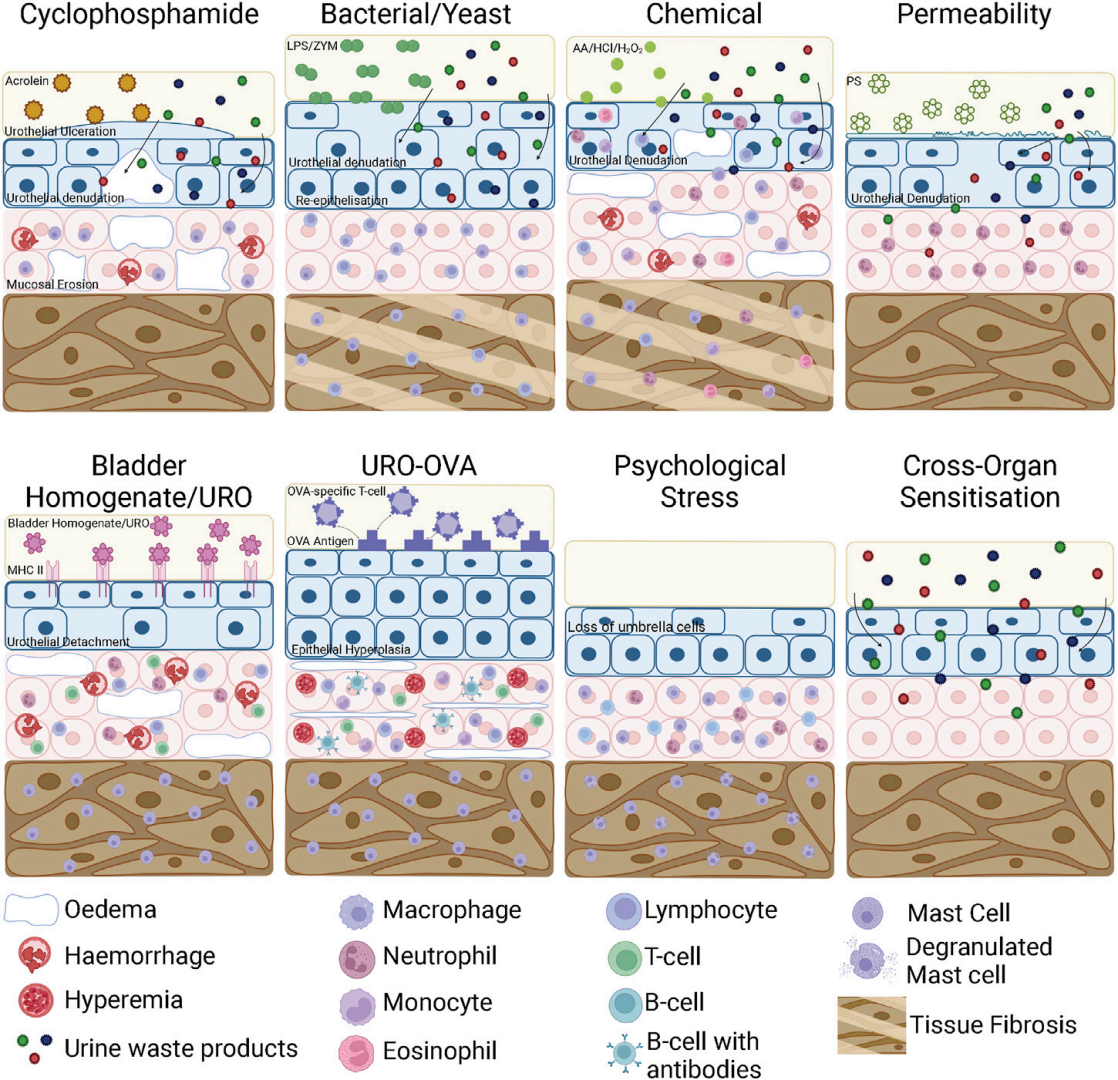
Bladder inflammation and damage in bladder cystitis animal models. In a CYP model, acrolein in the urine interacts with the urothelium to initiate an inflammatory response characterised by urothelial denudation and increased permeability, ulceration, interstitial oedema, mucosal erosion, haemorrhage and mast cell infiltration. LPS and ZYM bind to receptors on the surface of the urothelium, initiating an inflammatory response that results in urothelial denudation and bladder permeability. In chronic models, the urothelium thickens due to re-epithelisation and the detrusor layer undergoes tissue fibrosis. Chemical cystitis models induced by AA/HCl/H_2_O_2_ directly damage urothelial cells leading to urothelial denudation and severe inflammation characterised by mast cell, lymphocyte, monocyte, neutrophil and eosinophil infiltration as well as oedema and haemorrhage. In chronic stages of chemical cystitis models, the detrusor undergoes tissue fibrosis. Bladder permeability models such as protamine sulfate disrupt the GAG layer, increasing urothelial permeability and inducing mild inflammation that allows toxic waste products contained within the urine to access the bladder wall. In EAC models using bladder homogenates or urothelial products, the bladder homogenates/urothelial products interact with the membrane bound MHC Class II molecule and triggers an autoimmune reaction. This consists of urothelial detachment from the mucosa, mucosal oedema, haemorrhage and the recruitment of mast cells to the detrusor and neutrophils, lymphocytes and T-cells to the mucosa. In transgenic EAC models, OVA specific T-cells are recognised by the OVA ‘self’ antigen expressed on the urothelium of URO-OVA mice. This triggers an autoimmune response including epithelial hyperplasia, mucosal oedema, hyperaemia, infiltrating mononuclear cells and the recruitment of mast cells to the mucosa and detrusor. In psychological stress models, there is a loss of superficial umbrella cells in the urothelium, increased inflammatory cell infiltration into the mucosa and mast cell degranulation. Cross-organ sensitisation models increase bladder permeability but do not induce inflammation. CYP, Cyclophosphamide; EAC, Experimental Autoimmune Cystitis; AA, Acetic Acid; HCl, Hydrochloric acid; H_2_O_2_, Hydrogen Peroxide; PS, Protamine Sulphate; LPS, lipopolysaccharide; ZYM, zymosan; TNBS, trinitrobenzene sulfonic acid; WAS, Water Avoidance Stress; NMS, Neonatal Maternal Separation; URO-OVA, urothelium-ovalbumin. Figure was created using Biorender.com.

*In vivo* bladder instillation of protamine sulphate is the most common method for specifically inducing urothelial permeability (Figure 3) (Lavelle et al., 2002; Shin et al., 2011; Hurst et al., 2015). Protamine sulphate promotes an increase in urothelial permeability by inactivating the sulphated polysaccharides of the GAG layer, increasing transcellular permeability of the urothelium and thus increasing absorption of urine solutes (Lasič et al., 2015). At low doses (1–10 mg/ml) protamine sulphate induces only mild urothelial damage, including urothelial sloughing and an increase in transcellular permeability that returns to normal over a period of 7 days (Lavelle et al., 2002; Meerveld et al., 2015). At higher doses (50 mg/ml), however, bladders have been shown to develop urothelial ulceration and infiltration of neutrophils into the mucosa (Soler et al., 2008). Whilst this goes beyond an isolated urothelial permeability model, it provides insight into IC/BPS pathophysiology by confirming that a significant increase in bladder permeability is able to induce bladder inflammation (Figure 4) (Soler et al., 2008). The impact of protamine sulphate on bladder function has not been reported, and the two studies that have assessed bladder sensory output have described contrasting results. A single low dose of protamine sulphate (1 mg/ml) was found to induce bladder afferent hypersensitivity *ex vivo* at 1 day post infusion (Grundy et al., 2020b). Afferent hypersensitivity was characterised by an increase in peak firing and decreased activation thresholds to bladder distension that returned to baseline by day 7 post infusion (Grundy et al., 2020b). In contrast, Stemler et al. reported protamine sulphate treated mice (10 mg/ml) had significantly blunted visceromotor responses (VMR) to bladder distension at noxious bladder distension pressures (Stemler et al., 2013), indicative of reduced peripheral sensory drive from the bladder into the spinal cord. A recent study utilising a cocktail mixture of chondroitinase ABC and heparanase III to deglycosylate the proteoglycans of the GAG layer as an alternate method of urothelial barrier disruption (Offiah et al., 2017) induces acute increases in c-fos immunoreactivity in the spinal cord, significant decreases in abdominal mechanical withdrawal threshold to von-Frey hair (VFH) probing, and a significant increase in micturition reflex excitability. However, increased pelvic sensitivity and voiding parameters returned to control levels by day 7 post treatment (Offiah et al., 2017), which corresponds with urothelial barrier recovery in low-dose protamine sulphate treated bladders.

### 7.2 Inflammatory models of IC/BPS

Most animal models of IC/BPS attempt to create an inflammatory phenotype. This has been achieved by irritating the bladder urothelium with chemicals, chemotherapeutics, bacterial products and fungal ligands, and via induced urothelial autoimmunity. Importantly, these inflammatory models are not used because they are thought to be part of the underlying pathophysiology of IC/BPS in humans, although this may be true in some specific cases of cystitis, but because they induce bladder inflammation which leads to bladder hypersensitivity and recapitulates the key symptoms of IC/BPS patients, including increased urinary frequency and pelvic pain.

#### 7.2.1 Irritant models of inflammation

##### 7.2.1.1 Cyclophosphamide

Cyclophosphamide (CYP) is the most frequently used agent to induce cystitis in rodents. CYP is a chemotherapeutic for B cell malignant diseases and some solid tumours (Lee et al., 2014). A common and debilitating side effect of CYP treatment in humans is the development of chronic bladder inflammation and haemorrhagic cystitis, mimicking the most severe phenotypes of IC/BPS. CYP is metabolised to acrolein in the liver, a highly reactive aldehyde which is then renally excreted into the bladder (Figure 3) (Lee et al., 2014). While it accumulates in the bladder, acrolein interacts with the umbrella cells of the luminal urothelium, inducing an inflammatory response (Figure 4) (Lee et al., 2014). Both acute and chronic CYP dosing regimens have been used to generate cystitis in rodents, inducing urothelial permeability and a hypersensitive state characterised by altered voiding parameters and pelvic hypersensitivity (Hu et al., 2003; Chopra et al., 2005; Juszczak et al., 2010; Boudes et al., 2011; Auge et al., 2013; DeBerry et al., 2014; DeBerry et al., 2015; Auge et al., 2020; Chen et al., 2020; Yang et al., 2021; Yoshimura et al., 2021).

###### 7.2.1.1.1 Acute CYP treatment

Acute CYP treatment in rodents consists of a single high (150–200 mg/kg) dose injected intraperitoneally which leads to severe inflammation and dramatic alterations to bladder tissue morphology, bladder overactivity, and acute pelvic pain within 24 h (Boucher et al., 2000; Malley and Vizzard, 2002; Hu et al., 2003; Chopra et al., 2005; Smaldone et al., 2009; Juszczak et al., 2010; Auge et al., 2013; Okinami et al., 2014; Coelho et al., 2015; Chen et al., 2020). Bladder damage and inflammation following acute CYP treatment is characterised by a thickening of the bladder wall, mucosal erosion on the luminal surface of the urothelium, severe oedema, redness, ulceration of the urothelium and haemorrhage. This severe tissue damage is associated with infiltration of inflammatory cells (Yoshimura and de Groat, 1999; Boucher et al., 2000; Malley and Vizzard, 2002; Hu et al., 2003; Chopra et al., 2005; Smaldone et al., 2009; Juszczak et al., 2010; Auge et al., 2013; Chen et al., 2020), and elevated inflammatory cytokines, including IL-α, IL-1β, IL-2, IL-4, IL-5, IL-6, IL-10, IL-18, TNF-α/β, as well as the chemokine MCP-1 in the bladder wall (Malley and Vizzard, 2002; Smaldone et al., 2009; Auge et al., 2013; Jhang and Kuo, 2016a; Chen et al., 2020). Acute-CYP treatment in rats also significantly reduces transepithelial resistance and increases urothelial permeability to water and urea (Chopra et al., 2005).

Acute CYP treatment induces marked bladder hyperreflexia, characterised by an increase in non-voiding contractions and micturition frequency, and a decrease in the intercontraction interval and maximum voided volume for up to 36 h post CYP administration (Hu et al., 2003; Chopra et al., 2005; Juszczak et al., 2010; Okinami et al., 2014; Chen et al., 2020). Acute CYP treatment also results in a decreased nociceptive threshold in response to innocuous mechanical stimulation of the peritoneum with von Frey hairs *in vivo* in the 4 hours post CYP, and increased bladder afferent responses to distension *ex vivo* 24 h after CYP (Auge et al., 2013; Mills et al., 2020).

###### 7.2.1.1.2 Chronic cyclophosphamide treatment

Chronic dosing regimens involve repetitive doses of CYP at lower concentrations (40–100 mg/kg), given every 2–3 days for 7 or 10 days (Yoshimura and de Groat, 1999; Malley and Vizzard, 2002; Hu et al., 2003; Chopra et al., 2005; Dang et al., 2008; Juszczak et al., 2010; Boudes et al., 2011; Boudes et al., 2013; DeBerry et al., 2014; DeBerry et al., 2015; Gao et al., 2015; Sugino et al., 2015; Auge et al., 2020; Yang et al., 2021; Yoshimura et al., 2021), and have an added advantage of more closely mimicking a cyclophosphamide chemotherapy dosing schedule.

Like acute high-dose CYP, chronic CYP treatment in rats induces severe bladder inflammation including extensive mucosal erosion, ulcerations, oedema, occasional petechial haemorrhages and irregular ectasia vessels (Figure 4) (Malley and Vizzard, 2002; Hu et al., 2003; Juszczak et al., 2010; DeBerry et al., 2014; Gao et al., 2015; Sugino et al., 2015; Yoshimura et al., 2021). Inflammation is characterised by an increase in the number of inflammatory cells, including mast cells infiltrating the bladder mucosa (Malley and Vizzard, 2002; Juszczak et al., 2010; Yang et al., 2021; Yoshimura et al., 2021), and significant upregulation of inflammatory cytokines including IL-1β, IL-2, IL-6, IL-10 and TNF-α.

Chronic CYP treated rodents show increased sensory innervation within the bladder wall, hypersensitivity of bladder sensory nerves, and allodynia and hyperalgesia to VFH probing of the abdomen (Boudes et al., 2011; Auge et al., 2020). Chronic CYP-treated mice also develop an exaggerated VMR to noxious bladder distension (DeBerry et al., 2014) and increased pERK immunoreactivity in the lumbosacral dorsal horn following bladder distension (DeBerry et al., 2014), indicating there is an increased peripheral drive into the spinal cord due to exaggerated peripheral sensory signalling. These changes in bladder sensation and sensory signalling are also associated with significant alterations in cystometric parameters characteristic of an inflammatory phenotype (Yoshimura and de Groat, 1999; Dang et al., 2008; Boudes et al., 2013), including increased voiding frequency, decreased voided volume, reduced intercontraction intervals and increases in mean basal pressure compared to control animals (Dang et al., 2008; Juszczak et al., 2010; Yang et al., 2021; Yoshimura et al., 2021). Changes in bladder function and sensitivity evoked by chronic CYP treatment have been shown to persist long after treatment but most studies see the effects of treatment return to baseline by 7–10 days post treatment, highlighting the relative transience of even chronic CYP models. Whilst this is longer than the 1–3-day window of hypersensitivity that is induced by acute CYP dosing, it does not recapitulate the chronic and reportedly progressive nature of IC/BPS. CYP is also known to have global impacts on health, including body condition, weight loss, gastrointestinal dysfunction, and stress amongst others, yet the impacts of these extra-bladder effects are rarely considered when interpreting data from CYP animal models.

##### 7.2.1.2 Chemical cystitis

Rodent models of acute and chronic cystitis have also been induced by intravesical instillation of a variety of chemicals including hydrochloric acid (HCl), hydrogen peroxide (H_2_O_2_) and acetic acid (AA) (Figure 3).

Induction of chemical cystitis in animals using AA, HCl or H_2_O_2_ leads to severe bladder damage and inflammation (Figure 4). During the early stages of cystitis induction (1–3 days after chemical instillation), the bladder shows denudation of urothelial umbrella cells and an increase in urothelial permeability (Hauser et al., 2009). There is also the appearance of haemorrhage and oedema, and the infiltration of inflammatory cells throughout the bladder including neutrophils, monocytes, lymphocytes and natural killer cells (Dogishi et al., 2017; Song et al., 2017; Sahiner et al., 2018). In the later stages of cystitis progression, 7–14 days after chemical instillation, bladder damage becomes more severe with large fibrotic patches, vascular congestion and submucosal oedema as well as infiltration of chronic inflammatory cells including eosinophils and mast cells (Kirimoto et al., 2007; Dogishi et al., 2017; Danacioglu et al., 2021). By day 14, due to epithelial denudation, the bladder has significantly increased tissue fibrosis which leads to thickening of the bladder wall and hyperplasia (Song et al., 2015; Dogishi et al., 2017).

Intravesical AA in rats causes immediate bladder dysfunction, with reduced intercontraction intervals and bladder compliance 20–30 min after infusion that can persist for up to 2 weeks (Fraser et al., 2003). H_2_O_2_ instillation also leads to significantly more frequent micturition events and decreased voided volume after 24 h (Dogishi et al., 2017). Significantly shorter mean intercontraction intervals and the development of irregular non-voiding bladder contractions are also seen after bladder instillation of HCl or AA in rats (Çayan et al., 2003; Kirimoto et al., 2007; Song et al., 2015; Dogishi et al., 2017; Song et al., 2017). These effects are maintained 7–14 days after chemical instillation of HCl, AA or H_2_O_2_, highlighting the development of a persistent disease phenotype. Despite these well characterised changes in bladder function, reports of altered bladder sensation and or sensory signalling are lacking from preclinical studies.

#### 7.2.2 Bacterial and yeast products

Bacterial and fungal infections of the urinary tract are natural causes of bladder inflammation. To model this natural inflammation, a variety of studies have instilled biological inflammogens into the bladder, including Lipopolysaccharide (LPS), a bacterial product found in the outer membrane of gram-negative bacteria*,* and Zymosan, a ligand found on the surface of fungi, like yeast (Figure 3). In contrast to chemical irritants which rely on extensive tissue damage to induce inflammation, natural inflammogens produce a receptor-mediated reactive cellular process to induce inflammation. Zymosan binds to Toll-like receptor 2 (TLR2) and dectin 1 (Underhill et al., 1999; Sato et al., 2006), whilst LPS is detected by a diverse repertoire of proteins, including Toll-like receptors (TLRs), integrins, G-protein coupled receptors (GPCRs), and proteases (Kagan, 2017).

##### 7.2.2.1 Bacterial products

Lipopolysaccharide (LPS) has been commonly used to induce cystitis via direct infusion into the bladder for up to 45 min (Jerde et al., 2000; Lv et al., 2012; Sinanoglu et al., 2014; Li et al., 2017; Ryu et al., 2019; Yoshizumi et al., 2021). Protamine sulphate is often instilled into the bladder prior to LPS to weaken the GAG layer and allow LPS to interact directly with the urothelium (Lv et al., 2012; Sinanoglu et al., 2014; Li et al., 2017; Ryu et al., 2019). Modifications to the concentration, dwell time, and frequency of LPS instillations has allowed the development of both acute and chronic cystitis models.

Instillation of LPS in rodents induces bladder inflammation and bladder damage that persists up to 5 days after a single instillation. This includes severe disruption of bladder submucosal structures, vacuolisation of urothelial cells, infiltration of mononuclear and polymorphonuclear leukocytes and neutrophils and increased expression of pro-inflammatory cytokines IL-1α, IL-1β, IFN and TNF-α 24 h after a single LPS instillation (Jerde et al., 2000; Saban et al., 2002; Lv et al., 2012; Sinanoglu et al., 2014; Tambaro et al., 2014; Li et al., 2017). Bladder damage and inflammation is correlated with altered bladder voiding behaviour, increased intra-bladder pressure in response to bladder filling and shorter intercontraction intervals at 1–3 days post LPS instillation (Tambaro et al., 2014; Song et al., 2017). A single combined treatment of protamine sulphate and LPS leads to a significantly higher micturition frequency, as well as a significantly lower maximum storage pressure compared to that of controls 5 days after instillation in rats (Li et al., 2017).

To develop a more chronic cystitis phenotype LPS is instilled into bladders more than once, either daily for 4–14 days, twice weekly for 5 weeks, or once weekly for 4 weeks (Saban et al., 2007; Ryu et al., 2019; Chen et al., 2021; Yoshizumi et al., 2021). Chronic treatments result in a severely compromised urothelium that eventually leads to bladder remodelling caused by abnormally thick re-epithelialisation and tissue fibrosis (Figure 4) (Ryu et al., 2019; Chen et al., 2021; Yoshizumi et al., 2021). Inflammatory cells including macrophages, lymphocytes and mast cells infiltrate the bladder mucosa and enhanced cytokine concentrations are found in the urine (Saban et al., 2007; Ryu et al., 2019; Chen et al., 2021; Yoshizumi et al., 2021). At least 7 days after the final LPS instillations rodents exhibit bladder dysfunction characterised by shorter voiding intervals, increased non voiding contractions, decreased bladder capacity and significantly decreased peak and void threshold pressures compared to control animals (Song et al., 2017; Ryu et al., 2019; Chen et al., 2021; Yoshizumi et al., 2021). Additionally, chronic LPS-treated rats have increased pelvic hypersensitivity after 7, 14 and 21 days, with withdrawal thresholds to von-Frey hair probing of the abdomen and hind paw significantly decreased compared to controls (Yoshizumi et al., 2021) suggestive of altered bladder sensory signalling and pain processing.

##### 7.2.2.2 Zymosan

Various concentrations and dosing schedules of intrabladder zymosan infusion have been utilised to develop animal models of IC/BPS. The first reported use of zymosan as a model of cystitis employed a single intrabladder infusion of zymosan in rats. Rats developed an enhanced VMR to bladder distension and Evans blue extravasation in the bladder 24 h post infusion indicative of enhanced bladder permeability and bladder hypersensitivity to distension (Randich et al., 2006a; Randich et al., 2006b; DeBerry et al., 2007). Zymosan has also been shown to exaggerate the VMR to bladder distension in mice 24 h post infusion (Clodfelder-Miller et al., 2022). Combining zymosan with protamine sulphate (10 mg/ml) pre-treatment has recently been described in adult guinea pigs and was shown to induce inflammation and mucosal thickening 24 h post-infusion, sensitise bladder afferents to mechanical stimuli, and enhance the VMR to high pressure bladder distensions and increase voiding frequency (Ramsay et al., 2023).

Separate studies have investigated the long-term impact of zymosan induced cystitis utilising neonate rats. Epidemiological data indicates that inflammation in early life has correlative if not causative relevance to the pathophysiology of IC/BPS (Sharma et al., 2023). As such, this model was designed to recapitulate the real-world experience of early in life bladder inflammation and its potential to induce chronic changes in bladder sensation and function. Daily intrabladder instillation of zymosan in neonatal (p-14-18) rats resulted in bladder hypersensitivity in response to the infusion of ice-cold saline, increased sensitivity to intravesical potassium infusion and enhanced the VMR to bladder distension (Randich et al., 2006a; Randich et al., 2009; DeBerry et al., 2010; Ness et al., 2021). Neonatal zymosan also increased neurogenic plasma extravasation in the bladder and urinary frequency as adults, indicating the development of chronic changes in urothelial permeability and bladder sensitivity (Randich et al., 2006a; Randich et al., 2009; DeBerry et al., 2010; Ness et al., 2021). Intriguingly, no obvious histological abnormalities/damage are reported between neonatal zymosan, and control treated rats, suggesting changes in bladder function and sensitivity might be ‘locked in’ even following the resolution of zymosan induced inflammation in neonates (DeBerry et al., 2010). In mice, following the same neonatal zymosan treatment paradigm, neonatal bladder inflammation increases voiding frequency, and decreases intercontraction interval during cystometry. Neonatal bladder inflammation in mice did not evoke enhanced VMR to bladder distension suggesting species and/or mouse strains may be a defining feature in zymosan induced chronic hypersensitivity (Clodfelder-Miller et al., 2022).

Further exploration of the zymosan cystitis model has employed dual insults in both mice and rats, with neonatal multi-day zymosan being followed by a single zymosan infusion in adults. In general, the effect of this dual insult on cystitis induction is greater when compared directly to a single neonatal or adult insult, with animals exhibiting greater changes in bladder function and hypersensitivity. Rats and mice were reported to have decreased maximum micturition pressure and micturition weight as well as a significantly greater VMR to bladder distension (Randich et al., 2006a; Ness et al., 2021; Clodfelder-Miller et al., 2022), enhanced detrusor EMG responses to rapid increases in bladder pressure and enhanced abdominal withdrawal reflex scores 2–3 days after the final treatment as adults (Liu et al., 2021).

### 7.3 Autoimmune cystitis models

Experimental autoimmune cystitis (EAC) models have been developed based on the hypothesis that IC/BPS is caused by out-of-control bladder autoimmunity (Akiyama et al., 2020). Several methods of stimulating autoimmunity within the bladder wall have been explored.

#### 7.3.1 Urinary bladder homogenate

Subcutaneous injection of bladder homogenates into mice can induce EAC by making the bladder a target for autoimmunity (Figure 3). Briefly, bladders from female mice are homogenised and then lyophilised before being used to immunise recipient mice (Lin et al., 2008; Jin et al., 2017).

Urinary bladder homogenates have been used to induce EAC in both SWXJ and C57BL/6J mice strains (Lin et al., 2008; Jin et al., 2017; Liu et al., 2019). SWXJ strains are genetically more susceptible to the development of several autoimmune diseases initiated by Th1-type responses (Lin et al., 2008). However, immunising mice with bladder homogenates leads to the induction of EAC in both strains, but dual immunisation of bladder homogenates made 2 weeks apart was required to induce EAC in C57BL/6J mice. EAC was characterised by mast cell, neutrophil, and lymphocyte infiltration and significantly elevated levels of cytokines and chemokines within the bladder wall (Lin et al., 2008; Singh et al., 2013; Lin et al., 2008; Jin et al., 2017; Liu et al., 2019). Bladders developed submucosal oedema, urothelial detachment from the lamina propria, and mucosal thickening (Figure 4) (Lin et al., 2008).

Both SWXJ and C57BL/6 mice receiving subcutaneous injection of bladder homogenates develop bladder dysfunction in the form of shorter voiding intervals, decreased voided volume, and increased number of urine spots that were of a smaller size indicative of urinary frequency (Lin et al., 2008; Jin et al., 2017; Liu et al., 2019). Mice also developed hyperalgesia to von-Frey hair probing of the pelvic area and a decreased pelvic pain threshold 2 weeks after the second immunisation (Jin et al., 2017; Liu et al., 2019). To date, no assessment of bladder sensory nerve signalling or evoked pain responses such as VMR have been performed using this EAC model.

#### 7.3.2 Uroplakin models

Uroplakins (UP-Ia, UP-Ib, UP-II and UP-III) are a family of transmembrane proteins that are exclusively expressed in the bladder urothelium (Altuntas et al., 2012). Uroplakin EAC models are induced by subcutaneous injection of recombinant mouse uroplakin proteins that induce a urothelial-mediated cystitis (Figure 3).

Uroplakin EAC induced in SWXJ and BALB/c mice using rmUPK2 and UPK3A 65-84 respectively leads to extensive perivascular leukocyte (predominantly CD3^+^ T cells) infiltration (Altuntas et al., 2012; Izgi et al., 2013) and significantly elevated expression of genes encoding inflammatory cytokines TNF-α, IL-17A, IFN-γ, and IL-1β within the bladder (Altuntas et al., 2012; Izgi et al., 2013). A separate study also found significantly higher expression of the mast cell chemoattractant/activator CCL2 in the bladders of EAC mice; a contributing factor to the increased numbers of activated, resting and total mast cells in the bladder detrusor at up to 40 days after EAC induction (Bicer et al., 2015). Uroplakin EAC mice developed altered bladder function indicative of cystitis 5 weeks after immunisation, including increased urinary frequency and significantly decreased voided volume (Altuntas et al., 2012; Izgi et al., 2013). UPK3A 65-84 mice also showed greater sensitivity to von-Frey probing of the suprapubic region from day 5 after immunisation (Izgi et al., 2013; Bicer et al., 2015) that persisted for at least 40 days (Bicer et al., 2015). EAC induced in the rmUPK2 model did not demonstrate enhanced pelvic pain to noxious von-Frey forces (Altuntas et al., 2012). As different mouse strains as well as different recombinant mouse uroplakin proteins were used in these studies, it is not clear which is the fundamentally crucial variable. No assessments of bladder sensory nerve signalling or evoked pain responses such as VMR have been performed using this EAC model.

#### 7.3.3 Transgenic URO-OVA models

The most studied transgenic EAC mouse model is the URO-OVA model which requires the expression of the membrane form of model antigen ovalbumin (OVA) as a self-antigen on the bladder urothelium (Liu et al., 2007). This is achieved via the adoptive transfer of activated OVA-specific T-cells. These can either be OVA-specific CD8^+^ T cells from OT-I mice that express the transgenic CD8^+^ T-cell receptor specific for the OVA_257-264_ epitope peptide, or adoptive transfer of OVA-primed splenocytes after immunisation in C57BL/6 mice (Liu et al., 2007; Akiyama et al., 2021). These activated OVA-specific cells are then intravenously injected via the orbital sinus of URO-OVA mice to induce bladder cystitis (Figure 3) (Liu et al., 2007; Akiyama et al., 2021).

URO-OVA mice develop bladder inflammation from 7 days after adoptive T-cell transfer. Mice immunised with either OT-I or OVA-primed splenocytes developed interstitial oedema, increased vascularity, mononuclear cellular infiltration (predominantly T (CD3^+^) and B (CD19^+^) lymphocytes), mucosal hyperemia and epithelial hyperplasia (Figure 4) (Liu et al., 2007; Kim et al., 2011; Wang et al., 2016; Akiyama et al., 2021). However, no clear urothelial denudation was observed in the inflamed bladders (Akiyama et al., 2021). Increased mRNA expression of mast cell and sensory neuron-derived inflammatory factors MCP-1, IL-6, IFN-γ, TNF-α, NGF, pre-SP and substance P (Liu et al., 2007; Kim et al., 2011; Wang et al., 2016; Kullmann et al., 2018; Cui et al., 2019; Akiyama et al., 2021) as well as a two-fold increase in mast cells within the lamina propria and the detrusor of the bladder have been reported (Liu et al., 2007; Wang et al., 2016).

URO-OVA cystitis mice exhibit altered voiding behaviours characteristic of IC/BPS, including a decrease in maximum volume voided per micturition and an overall increase in the frequency of urination compared to control mice (Wang et al., 2016; Cui et al., 2019; Akiyama et al., 2021). URO-OVA cystitis mice also exhibited significantly decreased sensory thresholds to pelvic nociception (Cui et al., 2019; Akiyama et al., 2021) and an exaggerated VMR to bladder distension indicative of a pain phenotype (Cui et al., 2019). As with the previous models of autoimmune cystitis, no assessments of bladder sensory signalling have been performed in this EAC model.

### 7.4 Naturally occurring inflammatory models

Feline interstitial cystitis (FIC) is a naturally occurring idiopathic condition of domestic cats that exhibits physiological changes similar to those associated with IC/BPS without Hunner lesions (Birder et al., 2003; Kullmann et al., 2018; Mohamaden et al., 2019; Jones et al., 2021). FIC is broadly described as the presence of chronic, waxing and waning clinical signs of irritative voiding (pollakiuria, stranguria and dysuria) with an absence of neoplasia or bacteriuria, with or without the presence of uroliths or urethral plugs (Kruger et al., 1991; Kruger et al., 2009; Defauw et al., 2010; Lulich et al., 2010; Mohamaden et al., 2019; Jones et al., 2021). FIC exhibits similar histological features to IC/BPS without Hunner lesions including urothelial denudation or ulceration, submucosal oedema, chronic inflammatory cell infiltrates and muscularis fibrosis (Jones et al., 2021).

FIC cats have shown denudation and thinning of the urothelium (Lavelle et al., 2000), and urothelial spongiosis suggestive of a loss of cell-cell adhesion. Tight junctions were also found to have come apart leading to the disruption of the epithelial layer (Lavelle et al., 2000) and expression of E-cadherin tight junction protein was also significantly downregulated in FIC bladders compared with those of controls (Kullmann et al., 2018; Mohamaden et al., 2019). As a result, transepithelial resistance (TER) of the urothelium was significantly reduced, while water and urea permeability were significantly increased (Lavelle et al., 2000). Bladder oedema, haemorrhage, congestion of blood vessels, and infiltration of inflammatory cells in the bladder interstitium is also common in FIC (Kullmann et al., 2018; Mohamaden et al., 2019). FIC cats have been shown to have significantly higher serum concentrations of IL-1β, IL-6 and TNF-α compared to control cats (Mohamaden et al., 2019). Urine levels for IL-1β and IL-6, but not TNF-α were significantly higher than controls (Mohamaden et al., 2019), and a significant increase in mast cell number is observed in both the urothelium and lamina propria of FIC cats (Kullmann et al., 2018; Mohamaden et al., 2019).

Bladder Aδ afferents from FIC cats are hypersensitive to bladder distension (Roppolo et al., 2005), and urothelial cells from the bladders of FIC cats also demonstrate altered ATP release during cell swelling (Birder et al., 2003), which may contribute to additional activation or sensitisation of sensory afferent nerves (Birder and Andersson, 2013).

### 7.5 Psychological stress models

Chronic stress has been identified as a key factor in the development of IC/BPS, and early life stress has been shown to significantly increase the chances of developing chronic pelvic pain in later life (Fuentes and Christianson, 2018b). A large proportion of IC/BPS patients have experienced early-life adverse or traumatic events (Kullmann et al., 2018). Rodent psychological stress models are designed to recapitulate these human stressors via the induction of one or more stressful events that vary in their induction method and intensity. The most widely employed stress models to generate an IC/BPS phenotype are Water Avoidance Stress (WAS) or Neonatal Maternal-Separation (NMS).

#### 7.5.1 Water avoidance stress (WAS) models

WAS models are generated by exploiting the behavioural instincts of rats and mice, but is most commonly performed in rats (Chess-Williams et al., 2021; Gao and Rodríguez, 2022). A stressful environment is generated by placing rodents on a platform in the middle of a tank filled with water. Rodents will naturally avoid going into the water and, as there is no alternative way to escape, this raises systemic stress levels and leads to the development of anxiety (Figure 3). To establish a chronic stress phenotype, WAS is usually performed for 1 h a day over a period of 10 days (Robbins et al., 2007; Matos et al., 2017; Wang et al., 2017; Dias et al., 2019).

Wistar-Kyoto (WK) rats are commonly used for psychological stress models, as they are genetically predisposed to elevated levels of anxiety (Robbins et al., 2007). Compared to Sprague-Dawley (SD) rats, which are generally considered to be a low/moderate-anxiety strain, WK rats exhibit both neurochemical and behavioural differences in response to stress (Robbins et al., 2007). This includes a greater sensitivity to adverse events leading to an amplified HPA axis response and an attenuated brain noradrenergic system response (Robbins et al., 2007). WK, but not SD rats develop a significantly exaggerated VMR to urinary bladder distension following 10 consecutive days of WAS (Robbins et al., 2007).

WAS in WK rats results in a significant increase in urinary frequency, which includes a decrease in the average void size and an increase in the number of small voids as early as day 3 of the chronic WAS exposure protocol (West et al., 2021). Frequency of responses to von-Frey probing of the pelvic suprapubic area significantly increased during WAS from day 5 onwards to plateau on day 8 of treatment (Lee et al., 2015; Matos et al., 2017). WAS rats also develop altered bladder function, with cystometry revealing the voiding phase of micturition occurs at a significantly decreased pressure threshold (Wang et al., 2017; Gao et al., 2018). The VMR to urinary bladder distension is also evoked at a lower bladder pressure and for a longer duration in WAS rats compared to controls, representing a longer sensory/pain response to bladder stimuli (Wang et al., 2017; Gao et al., 2018). No assessments of bladder sensory signaling have been reported following WAS and so it is unclear if alterations in bladder sensitivity and function are due to changes in central processing circuits, sensitisation of peripheral sensory nerves, or both. Interestingly, the changes in bladder sensation and function observed during and following WAS also evoke changes in bladder physiology that show similarity to changes observed in humans (Chess-Williams et al., 2021), including loss of superficial umbrella cells leading to an altered urothelial surface, inflammatory cell infiltration into the mucosa, and mastocytosis (Figure 4) (Matos et al., 2017). WAS has also been frequently used as an animal model of irritable bowel syndrome (Moloney et al., 2016), a disorder characterised by chronic abdominal pain which commonly occurs with IC/BPS.

#### 7.5.2 Neonatal maternal separation (NMS) models

NMS models are generated by separating litters of pups from the dam for up to 3 weeks from postnatal day one, depriving the pups of innate needs and natural connection with their mother that imitates early-life trauma in humans (Figure 3). NMS induces chronic changes in bladder sensitivity and function in adult mice including increased voiding frequency with smaller void spots as compared to naïve mice (Pierce et al., 2018), and a significantly greater VMR in response to UBD at 8 weeks of age (Pierce et al., 2018).

The psychological nature of this model is shown in the effects NMS has on brain function. Mice exposed to NMS show significantly lower mRNA levels of corticotropin-releasing factor (CRF_1_) and glucocorticoid (GR), and higher brain-derived neurotrophic factor (BDNF) in the hippocampus (Pierce et al., 2018). As CRF is the main activator of HPA axis signalling, lower CRF and GR levels indicate decreased inhibition on the HPA axis (Pierce et al., 2018), which would explain the chronic changes in corticosterone response (Kalinichev et al., 2002; Nishi et al., 2014). This is hypothesised to have a cumulative effect, decreasing animal resilience to stress over the lifetime. As with WAS, a translation of psychological insult to physiological changes in the bladder occurs, with NMS mice having a significantly higher percentage of degranulated mast cells in the bladder (Figure 4) (Pierce et al., 2018).

To model the human experience more closely, whereby early life stress is commonly followed by numerous additional stressors, a psychological stress model has recently been generated that combines NMS followed by WAS at 8+ weeks of age (Pierce et al., 2016). Combining NMS and WAS impacted bladder function, as measured by a significant increase in the number of voids and a higher total urine output in mice 1d-post WAS that returned to baseline at 8d-post WAS (Pierce et al., 2016). Implementation of these combined models also led to an altered VMR to urinary bladder distension (Pierce et al., 2016). At 1d post-WAS, NMS mice exhibited a transient decrease in VMR during bladder distension which was later significantly increased at 8d post-WAS (Pierce et al., 2016) suggesting combined stressors may induce more longer lasting changes in bladder sensitivity than WAS alone.

### 7.6 Cross-organ sensitisation models

Considerable clinical evidence links diseases of the colon, such as irritable bowel syndrome (IBS) and inflammatory bowel disease (IBD), with IC/BPS (Grundy and Brierley, 2018). 20%–30% of both men and women with IC/BPS report IBS as among their most common comorbidity (Alagiri et al.; Clemens et al., 2006) and IC/BPS patients are 100 times more likely to have concurrent IBD than healthy controls (Alagiri et al.). More recently, it has been shown that having IBS increases the risk of developing IC/BPS (Chang et al., 2021), however, the underlying mechanisms responsible for the development of comorbid visceral pain syndromes have yet to be fully elucidated. Cross-sensitisation of the peripheral and central sensory pathways that co-innervate pelvic organs such as the bladder and colon has been proposed as a major contributing factor (Grundy and Brierley, 2018).

Animal models have been generated to recapitulate cross-organ sensitisation between the colon and bladder by inducing long term hypersensitivity of the sensory pathways that innervate the colon. By far the most well characterised of these methods is following intracolonic co-administration of ethanol and trinitrobenzene sulfonic acid (TNBS) (Figure 3). Ethanol is required to disrupt the intestinal barrier and enable the interaction of TNBS within the colon wall to elicit an immune response (Antoniou et al., 2016). The severity of colitis in these models is directly related to the doses of ethanol and TNBS, and shows significant variability between species (rat vs. mouse) as well as genotype (Antoniou et al., 2016). In general, the first week following a single TNBS/ethanol enema is characterised by severe colonic inflammation (Hughes et al., 2009; Vannucchi and Evangelista, 2018). By 7 days post-TNBS administration, colonic inflammation has begun to spontaneously resolve, with a corresponding increase in the integrity of the colonic wall (Hughes et al., 2009). By 28 days post-TNBS, there are no observable histological changes in the colon compared with healthy control mice, yet hypersensitivity of peripheral sensory pathways persists (Hughes et al., 2009). Whilst TNBS colitis has not been observed to induce marked inflammation in the bladder (Grundy et al., 2018d; Grundy and Brierley, 2018), studies in rats have demonstrated that urothelial permeability increases during the active phase of TNBS-induced colonic inflammation (0–7 days) in the absence of overt histological damage to the bladder (Figure 4) (Meerveld et al., 2015; Towner et al., 2015).

Mice and rats in the acute phase of TNBS colitis develop bladder hypersensitivity characterised by changes in micturition parameters and exaggerated bladder afferent sensitivity in the absence of overt changes in bladder histology (Lei and Malykhina, 2012; Xia et al.). Despite the resolution of colonic inflammation, reduced bladder capacity, voided volumes, and intermicturition intervals and changes in bladder voiding patterns indicative of bladder overactivity persist in both rats and mice up to 90 days post-TNBS (Lamb et al., 2006; Liang et al., 2007; Ustinova et al., 2007; Fitzgerald et al., 2013; Grundy et al., 2018d). TNBS colitis has also been shown to enhance the VMR to urinary bladder distension (Lamb et al., 2006) and induce hypersensitivity of bladder-innervating sensory nerves to bladder distension both during the active and post-inflammatory phase of TNBS colitis (Ustinova et al., 2006; Ustinova et al., 2007; Grundy et al., 2018d). Together, these studies show that experimental colitis can induce chronic changes in the sensory networks that regulate bladder sensory signalling and provide important information on the mechanisms that might underlie the development of IC/BPS in patients that have comorbid visceral pain disorders such as IBS.

## 8 Discussion

Animal models of human disease have two primary aims: to unravel the pathophysiological mechanisms that drive the development and maintenance of the disease, and the testing of therapeutics for clinical translation. Developing a single animal model of IC/BPS to achieve these aims has been a major challenge, as there is significant heterogeneity in the pathological presentation of IC/BPS patient cohorts. As such, effective clinical translation may very well depend on the degree of progress made towards determining the mechanisms underlying the development of IC/BPS in patient cohorts. As these knowledge gains have an unknown timeline, researchers have in the interim developed a large variety of animal models that focus on specific aspects of known IC/BPS pathophysiology. Validation of these models for relevance to IC/BPS and therapeutic translation has primarily focused on recapitulating the major clinical symptoms seen in IC/BPS patients.

### 8.1 Recapitulating IC/BPS symptoms

Chronic pelvic or bladder pain are the defining and most debilitating symptoms of IC/BPS. IC/BPS patients display hypersensitivity to bladder distension that translates into increased pain during bladder filling and the development of lower urinary tract symptoms including increased urinary urgency and frequency (Hanno et al., 2011a; Hanno et al., 2011b; Lai et al., 2015). Patients will also demonstrate mechanical hypersensitivity to noxious stimulation in the suprapubic area during sensory testing that reflects the development of referred hyperalgesia from the bladder (Lai et al., 2014; Warren, 2014). As such, it is essential that any animal model intended to be used in the preclinical evaluation of therapeutics for IC/BPS patients develops bladder and/or pelvic pain. Furthermore, as patients with IC/BPS have painful symptoms that persist indefinitely, and often develop additional comorbidities over time (Driscoll and Teichman, 2001), animal models of IC/BPS should ideally see the development of a pelvic/bladder pain phenotype that endures or even increases in intensity over time.

### 8.2 Pain and hypersensitivity

Significant progress has been made in developing animal models of IC/BPS that exhibit pelvic/bladder pain, including the development and validation of techniques to accurately assess evoked bladder pain and hypersensitivity. These include the VMR to measure abdominal contractions as a surrogate for bladder pain during bladder distension, von-Frey hair probing of the suprapubic region to assess referred hyperalgesia, and sensory nerve recordings during bladder distension to directly record sensory nerve output in response to bladder distension or stretch. Combinations of these techniques have been used extensively to characterise the development of an IC/BPS phenotype in the models presented in this review. The use of different techniques to assess bladder hypersensitivity and pain both across and within animal models reflects the diversity of experimental techniques available to individual research groups. However, as most models have now been validated by multiple research groups, a reliable assessment of each model has started to emerge (Table 2). Bladder permeability, cyclophosphamide, zymosan, lipopolysaccharide, autoimmune cystitis, feline interstitial cystitis, psychological stress, and cross-organ sensitisation models all report the development of evoked bladder hypersensitivity and/or pain reflective of an IC/BPS phenotype. The variability in the degree of bladder pain/hypersensitivity across these models is dependent on both the intensity of the sensitising stimulus as well as the method of induction. In bladder centric models, increased bladder pain/hypersensitivity is typically correlated with a greater severity of inflammation/permeability, with models that combine to simultaneously impact permeability and inflammation shown to impart greater effects on peripheral afferent sensitisation and pain signaling.

Psychological stress and cross-organ sensitisation models can induce both bladder pain and hypersensitivity in the absence of significant bladder inflammation. However, the intensity of the stimulus, including the duration or timing of psychological stress, or magnitude of the colonic insult, remains a crucial factor in determining whether animals develop measurable bladder pain/hypersensitivity. Furthermore, for psychological stress models, utilising specific rodent species and strains that are predisposed to anxiety are crucial for the successful development of bladder pain/hypersensitivity.

The evidence generated from these models supports comprehensive clinical data showing that the aetiology of IC/BPS can be highly diverse, with multiple mechanisms capable of contributing to the development of bladder centric symptoms. The ability to accurately recapitulate the major clinical features of IC/BPS in animal models also supports their continued use in unravelling the mechanisms responsible for the development and maintenance of IC/BPS symptoms. However, as described in detail for each model, the relative transience of bladder pain/hypersensitivity in many of these models remains a limiting factor in their utilisation for exploring novel therapeutics for IC/BPS. Furthermore, whilst these studies comprehensively characterise evoked pain in animal models of IC/BPS, experimental assessment of non-evoked pain, such as grimace scales, burrowing, and automated behavioural analysis that have been employed in a variety of other pain models is not commonly reported (Deuis et al., 2017). As IC/BPS patients report both evoked pain relating to bladder filling as well as non-evoked pain, incorporating experimental techniques that assess non-evoked pain into future research designs may be useful in fully characterising the clinical translatability of IC/BPS animal models. Non-evoked pain assessments are primarily observation based and therefore have the advantage that they can be easily incorporated into an experimental design without the need for additional animal cohorts or specialised equipment.

### 8.3 Chronicity

Although many animal models accurately recapitulate the evoked bladder pain and hypersensitivity phenotype of IC/BPS, animal models that develop a chronic pain phenotype are scarce. In the most explored bladder-centric permeability and inflammatory models, pain and/or hypersensitivity effects are commonly reported only in the day/s, or first week after ceasing treatment. Whether this is due to not exploring more chronic time points or reflects a resolution of hypersensitivity and pain is not always clear, but it is of paramount importance, as IC/BPS is by definition a chronic disorder. As a result, the last decade has seen a discernible drive to refine preclinical models of IC/BPS to induce a more chronic pain state. This has been achieved by modulating the strength, timing, and duration of the model induction, such as with multiple lower doses of cyclophosphamide administered over a longer period, several LPS administrations over a longer time course, neonatal insult, or chronic exposure to stressful stimuli during a specific developmental phase. These refinements will likely prove crucial in developing the next-generation of treatments for IC/BPS, as they provide greater opportunities to interrogate the mechanisms underlying long term changes in bladder sensation.

Further development of IC/BPS animal models has revealed neonatal maternal stress, neonatal zymosan, and colon-bladder cross-sensitisation models exhibit bladder hypersensitivity and pain long after the resolution of the initial sensitising stimulus and in the absence of overt bladder damage. This is crucial, as it reveals that chronic changes in bladder sensory pathways can occur to embed a hypersensitive state long after an initiating stimulus. Many IC/BPS patients present without obvious bladder pathophysiology at the time of diagnosis, but have a history of chronic psychological stress, urinary tract infections, or comorbidities including IBS. As such, the development of animal models that see a chronic pelvic/bladder pain phenotype in the absence or following resolution of inflammation represents a crucial step towards accurate pre-clinical modelling of these IC/BPS patient subsets.

A logical next step in the development of chronic IC/BPS models is to expand the dual insult models established using intrabladder zymosan to include heterogenous elements of distinct animal models. This may include combining an early in life event, such as neonatal inflammation to mirror childhood UTI, followed by chronic stress in adulthood or visa-versa.

### 8.4 Translational potential

The ability to accurately recapitulate the major symptoms of IC/BPS in animal models is a crucial step towards the development of novel therapeutics for IC/BPS. However, the variety of animal models available that develop an IC/BPS like phenotype, and the stark differences in induction methods, raises important questions as to which model is most suited for the pre-clinical evaluation of therapeutics for future clinical translation. Consideration must be given to the pathophysiological origin, the type/severity of model, and the time after model induction that assessments are made.

A balance must be sought between how closely an animal model of IC/BPS shares pathophysiological traits with IC/BPS patients, and model presentation. For example, chemical models such as acetic/hydrochloric acid and hydrogen peroxide induce a well characterised cystitis phenotype. However, chemical cystitis methods appear limited in their translational potential by an artificial method of induction that lacks an obvious link to the pathophysiology of IC/BPS. In contrast, models of urothelial permeability induce an acute IC/BPS phenotype that closely mirrors the presentation of many IC/BPS patients with a diminished urothelial barrier. However, as is well documented, in the absence of inflammation, the urothelial barrier is rapidly restored, and bladder hypersensitivity normalises, undermining its relevance to studying the chronic nature of IC/BPS. Current studies indicate zymosan and LPS models may represent a good choice for investigating an inflammatory IC/BPS phenotype. Both LPS and zymosan induce an IC/BPS like phenotype characterised by bladder inflammation and bladder pain. However, in contrast to chemical cystitis, inflammation is induced via the recruitment of natural inflammatory pathways associated with receptor activation rather than direct tissue damage. By more closely mirroring the natural inflammatory pathways reported to be activated in the bladders of IC/BPS patients, these models appear to have greater translational potential for investigating therapies that target bladder inflammation and/or inflammation induced hypersensitivity. Experimental autoimmune cystitis (EAC) mice also develop IC/BPS like symptoms, but whilst the inflammation is initiated by a cellular mediated mechanism, the severity is beyond the pathophysiology characterised for most IC/BPS patients. However, as some evidence supports an autoimmunological inflammatory process as an underlying contributor to pathophysiology in IC/BPS with Hunner lesions (Akiyama et al., 2020), EAC models may represent the best choice for investigating novel therapies for this severe IC/BPS phentypes.

The translational potential of an animal model also requires consideration of the mechanism of action of any proposed intervention in relation to the pathology that is thought to underlie the development of IC/BPS symptoms in a specific patient cohort. For instance, bladder hypersensitivity and pain in psychological stress models is caused by chronic dysregulation of the HPA axis. As such, preclinical evaluation of therapies that have peripheral sites of action, such as direct bladder infusions or anti-inflammatory agents are unlikely to be as efficacious as those that are tailored to central stress mechanisms such as psychotherapy, exercise, or bladder training. Similarly, targeting the HPA axis to treat IC/BPS symptoms that develop due to localised bladder inflammation and the sensitisation of bladder-innervating sensory nerves is unlikely to be as effective as directly targeting the bladder or peripheral sensory pathways. Exploring therapies in animal models of cross-organ sensitisation or early in life intervention will likely need a different approach entirely, as the pathophysiology of these disorders is embedded within the chronic remodelling of peripheral and central sensory circuits that establishes a chronically sensitised state. Considering these factors in the design of clinical trials for IC/BPS to aid patient stratification would also have major implications for interpreting success/failure of a particular intervention.

## 9 Conclusion

Significant progress continues to be made in the development of animal models of IC/BPS that more closely mimic the human condition. At present it appears unlikely that animal models will ever be able to perfectly model IC/BPS, owing to both symptom and pathophysiological heterogeneity amongst patients. However, as described in this review, multiple animal models of IC/BPS are now able to accurately reflect the major symptoms of IC/BPS including bladder hypersensitivity and pain. Whilst pre-clinical models need to be refined further to unravel the pathological mechanisms underlying the development of IC/BPS, being able to assess impacts of interventions on the primary symptoms of IC/BPS should hopefully pave the way for the development of novel therapeutics. However, with a large variety of distinct animal models available, care must be taken to select the appropriate model to ensure potential pre-clinical translation is maximised. Therefore, it is likely that different models will continue to be required for pre-clinical drug development based on the unique IC/BPS aetiology under investigation.
